# Reactions of hypobromous acid with dimethyl selenide, dimethyl diselenide and other organic selenium compounds: kinetics and product formation†

**DOI:** 10.1039/d3ew00787a

**Published:** 2024-01-08

**Authors:** Emanuel Müller, Urs von Gunten, Julie Tolu, Sylvain Bouchet, Lenny H. E. Winkel

**Affiliations:** a Swiss Federal Institute of Aquatic Science and Technology, Department of Water Resources and Drinking Water (W+T), Eawag Ueberlandstrasse 133 CH-8600 Duebendorf Switzerland lenny.winkel@eawag.ch +41 58 765 5601; b Swiss Federal Institute of Technology, Institute of Biogeochemistry and Pollutant Dynamics (IBP), Department of Environment Systems (D-USYS), ETH Zurich Universitätsstrasse 16 8092 Zürich Switzerland; c School of Architecture, Civil and Environmental Engineering (ENAC), École Polytechnique Fédérale de Lausanne (EPFL) 1015 Lausanne Switzerland

## Abstract

Selenium (Se) is an essential micronutrient for many living organisms particularly due to its unique redox properties. We recently found that the sulfur (S) analog for dimethyl selenide (DMSe), *i.e.* dimethyl sulfide (DMS), reacts fast with the marine oxidant hypobromous acid (HOBr) which likely serves as a sink of marine DMS. Here we investigated the reactivity of HOBr with dimethyl selenide and dimethyl diselenide (DMDSe), which are the main volatile Se compounds biogenically produced in marine waters. In addition, the reactivity of HOBr with further organic Se compounds was tested, *i.e.*, SeMet (as *N*-acetylated-SeMet), and selenocystine (SeCys_2_ as *N*-acetylated-SeCys_2_), as well as the phenyl-analogs of DMSe and DMDSe, respectively, diphenyl selenide (DPSe) and diphenyl diselenide (DPDSe). Apparent second-order rate constants at pH 8 for the reactions of HOBr with the studied Se compounds were (7.1 ± 0.7) × 10^7^ M^−1^ s^−1^ for DMSe, (4.3 ± 0.4) × 10^7^ M^−1^ s^−1^ for DMDSe, (2.8 ± 0.3) × 10^8^ M^−1^ s^−1^ for SeMet, (3.8 ± 0.2) × 10^7^ M^−1^ s^−1^ for SeCys_2_, (3.5 ± 0.1) × 10^7^ M^−1^ s^−1^ for DPSe, and (8.0 ± 0.4) × 10^6^ M^−1^ s^−1^ for DPDSe, indicating a very high reactivity of all selected Se compounds with HOBr. The reactivity between HOBr and DMSe is lower than for DMS and therefore this reaction is likely not relevant for marine DMSe abatement. However, the high reactivity of SeMet with HOBr suggests that SeMet may act as a relevant quencher of HOBr.

## Abstract 2

We studied reactions between a variety of organic forms of the essential element selenium (Se) and the oxidant hypobromous acid (HOBr). The studied Se compounds, especially selenomethionine, had a high reactivity with HOBr.

## Graphical abstract

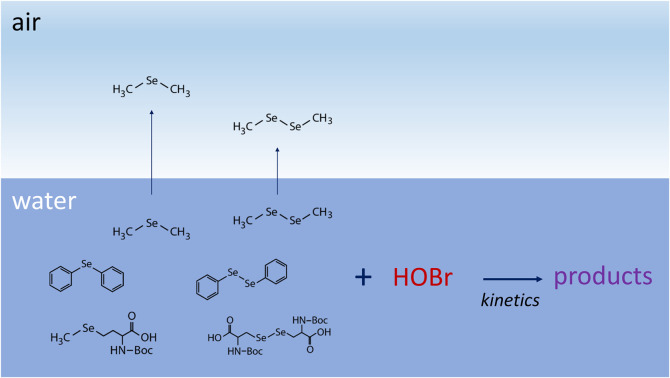

**Water impact**

Selenium is an essential element in low doses but toxic at higher levels. It can be found in natural and engineered aquatic systems in organic forms and potentially react with the important oxidant hypobromous acid (HOBr). Our study, focusing on seawater, shows that selected organic selenium compounds indeed react fast with HOBr and may thus act as relevant quenchers for reactive bromine in seawater and other aquatic systems.

## Introduction

Selenium (Se) is an essential micronutrient for many living organisms, including mammals, bacteria and archaea, and some species of microalgae, while the essentiality of Se for plants is still controversial.^1–4^ Multiple biological functions and beneficial effects on organisms' health are attributed to Se, based on the redox activity of Se compounds. Undersupply of Se has been associated with various human health issues, including cardio-myopathy (Keshan disease), cancer, immune and endocrine disfunction, muscle and bone disorder, neurodegenerative diseases, decreased thyroid function and male infertility.^5,6^ Where in many areas bedrock is low in Se, the atmosphere is an important source of Se to terrestrial food systems, mainly in the form of wet deposition.^7–10^ Marine environments play an important role in the atmospheric Se budget *via* biogenic emissions of the volatile organic compounds dimethyl selenide (DMSe) and dimethyl diselenide (DMDSe).^9,11^ In marine waters, DMSe was detected in concentrations ranging from 0.06 to 4.73 pM^11,12^ and its production was reported for macroalgae,^13^ marine bacteria^14,15^ and has been suggested for marine microalgae such as coccolithophorids (Table S1, ESI†).^11,12,16–18^ For DMDSe, which has been reported in concentrations between 0.02–0.26 pM Se in marine waters,^11,12^ the production pathways are largely unknown, although, various biotic/abiotic pathways have been suggested as well as different precursors (selenols, selenocysteine (SeCys), selenocystine (SeCys_2_) and selenomethionine (SeMet)).^19–22^

Recently, we reported a fast reaction (second-order rate constant: *k* = 1.6 × 10^9^ M^−1^ s^−1^) between the sulfur analog of DMSe, *i.e.*, dimethyl sulfide (DMS) and the marine oxidant hypobromous acid (HOBr), produced *via* the oxidation of bromide (Br^−^) by H_2_O_2_, catalyzed by the enzyme vanadium-bromoperoxidase (V-BrPO).^23^ Therefore, we concluded that this reaction is likely an important sink for marine DMS.^24,25^ Compared to organic sulfur, redox reactions for the analogous organic Se compounds generally have been reported to be faster, with 1–2 orders of magnitude higher second-order rate constants than for their analogous S compounds.^26^ Therefore, organic Se compounds may be effective oxidant scavengers, although, previous studies reported diselenides to be less efficient in scavenging hydrogen peroxide (H_2_O_2_) and singlet oxygen (^1^O_2_) than selenols and selenides.^27^ Furthermore, the Se amino acids SeCys (also referred as the 21st amino acid) and SeMet, are quickly oxidized to the diselenide “selenocystine (SeCys_2_)” and the selenoxide “Selenomethionine-oxide”, respectively, but can also be rapidly reduced back to SeCys and SeMet.^26,28–32^ In contrast to the higher reactivity of organic Se than organic S, it has been demonstrated that the inorganic Se compound selenite has an almost 6 order of magnitude lower second-order rate constant for the reaction with HOBr than sulfite, the inorganic S analog.^33^ The reason for this difference is currently unknown.

The main objective of this study was to determine apparent second-order rate constants for the reactions between HOBr and DMSe, and HOBr and DMDSe. Furthermore, the reactivities of HOBr with SeMet (as *N*-acetylated-SeMet), and SeCys_2_ (as *N*-acetylated-SeCys_2_) were investigated. These compounds are likely precursors of volatile organic Se in marine waters and organisms. We also investigated the kinetics of the reactions of HOBr with the aryl-analogs of DMSe and DMDSe, *i.e.*, diphenyl selenide (DPSe) and diphenyl diselenide (DPDSe), which are industrially produced organic Se compounds used in chemical synthesis and as green catalysts for medical and pharmacological purposes.^34–36^ These aromatic analogs of the aliphatic compounds DMSe and DMDSe, respectively, were selected to obtain further insights into the substitution effects of organo-Se compounds on the reactivity with HOBr. Furthermore, Se-containing oxidation products from the above reactions for different molar HOBr : Se compound ratios were identified and if possible quantified using high-resolution mass spectrometry (HR-MS) and liquid chromatography coupled to inductively coupled plasma mass spectrometry (LC-ICP-MS/MS). Finally, the reactivity of HOBr with the selected organic Se compounds was compared to the analogous S compounds.

## Materials and methods

### Chemicals and reagents

For treatment of glassware and handling of chemicals see Text S2, ESI.† A list with information of all used chemicals is provided in Table S2, ESI.†

#### Reagents for kinetic experiments

##### Se compounds

Stock solutions of dimethyl selenide (DMSe), dimethyl diselenide (DMDSe) and diphenyl selenide (DPSe) were produced in gastight 10 mL headspace amber crimp vials (ND20, 46 × 22.5 mm, BGB Analytics, Boeckten, Switzerland) by diluting the pure compound in methanol (0.25%; corresponding to *ca.* 25 mM of the Se compound concentration). Stock solutions of diphenyl diselenide (DPDSe), seleno-dl-methionine (SeMet) and seleno-l-cystine (SeCys_2_) were produced by weighing the required quantity of the solid material in a 10 mL headspace amber crimp vial and dissolving it either in ethanol or water (6 mM DPDSe in ethanol, 6 mM SeMet in water and 0.6 mM SeCys_2_ in water). Solutions of the volatile substances DMSe and DMDSe were produced daily, whereas the other Se stock solutions were produced monthly.

##### Acetylation of SeMet and SeCys_2_ amino groups

Di-*tert*-butyl dicarbonate (BOC_2_O) and sodium hydrogen carbonate (NaHCO_3_) were used to acetylate the amino groups of SeMet and SeCys_2_ to block them against reactions with HOBr (method described in Text S3, ESI†). The stability of the BOC_2_O-protection group for *N*-acetylated-SeMet and *N*-acetylated-SeCys_2_ was tested by chloramine formation during chlorination (see Table S2 and Text S4, ESI†).

##### HOBr

Stock solutions of HOBr were produced by mixing sodium hypochlorite (NaOCl) and potassium bromide (KBr), as described previously*.*^24^

##### Competitors for competition kinetics

Resorcinol and 1,3,5-trimethoxybenzene (TMB) were used as competitors for competition kinetics. A 250 mM resorcinol stock solution was produced in ultrapure water and further diluted to working concentrations of 2.5 mM and 250 μM. A saturated solution of TMB was produced in ultrapure water and its effective concentration was determined by a methanolic stock solution of known concentration (dissolved TMB concentration: 2.5 mM in water). Both resorcinol and TMB stock solutions were stored in 10 mL headspace amber crimp vials at 4 °C.

#### Buffer and standard solutions

Phosphate buffer solutions (40 mM PO_4,tot_, pH 8) and artificial seawater medium (0.55 M sodium chloride, NaCl; 840 μM potassium bromide, KBr) were used for kinetic experiments (for more details see ref. 24). NaHCO_3_ (1 mM, pH 8) was used as a buffer solution for Se-product analyses.

Potassium perchlorate (KClO_4_) was used to maintain constant ionic strength of the reaction solution. Perchlorate concentrations of 100 and 50 mM were used depending on the phosphate buffer concentration and solution pH.

Standard solutions of Se compounds and competitors were prepared freshly every day for the following calibration ranges: DMSe = 0–800 nM, DMDSe = 0–125 nM, DPSe, Res, TMB = 1.67–16.7 μM, DPDSe = 1–10 μM.

### Kinetic experiments and determination of second-order rate constants

#### Experimental procedure

To determine second-order rate constants for the reactions of DMSe, DMDSe, DPSe, *N*-acetylated-SeMet and *N*-acetylated-SeCys_2_ with HOBr, competition kinetics experiments were performed with resorcinol as a competitor (Table 1), which has a pH-dependent reactivity with HOBr (Table S4 and Fig. S2, ESI†). For the kinetic experiments with DPDSe, TMB was used as a competitor (Table 1). Experiments were performed in triplicate at pH 8, which represents the average ocean pH and is close to physiological conditions. The standard deviation of the replicates was taken as a base for the error calculation of second-order rate constants (Table S5, ESI†). We followed the protocol used in Müller *et al.* 2019 (ref. 24) for organic S compounds, but using lower concentrations of organic Se compounds and thus also of HOBr and phosphate buffer. For the kinetic experiments with DMSe, DMDSe, DPSe, *N*-acetylated-SeMet, the concentrations were as follows: phosphate buffer solution (pH 8), 20 mM; organic Se compound, 12.5 μM; and competitor, 12.5 μM. Kinetic experiments with *N*-acetylated-SeCys_2_ were performed with lower phosphate buffer and Se compounds concentrations, *i.e.*, 10 mM and 8 μM, respectively (competitor at 12.5 μM). For experiments with DPDSe, the concentrations were as follows: phosphate buffer (pH 8), 10 mM; DPDSe, 2.9 μM and competitor, 6.25 μM, respectively. No methanol (MeOH) was used for the experiments with *N*-acetylated-SeMet, while 0.5% MeOH was used for experiments with DMSe, DMDSe, DPSe, and 5% MeOH for DPDSe and *N*-acetylated-SeCys_2_. Owing to the relevance of DMSe and DMDSe in marine waters, kinetic experiments with DMSe and DMDSe were performed in buffered artificial seawater medium in addition to ultrapure water. Reactions were initiated by injecting HOBr in a volumetric 1 : 1 ratio, targeting doses of 0, 2.5, 5, 7.5, 10, 12.5, 15 μM HOBr for experiments with DMSe, DPSe and *N*-acetylated-SeMet; 0, 5, 10, 15, 20, 25 μM HOBr for experiments with DMDSe and *N*-acetylated-SeCys_2_; and 0, 2.5, 3.75, 5, 6.25, 7.5 μM HOBr for experiments with DPDSe. To guarantee fast and complete HOBr consumption, *i.e.*, within 1 min under magnetic stirring, all HOBr doses were lower than the sum of the concentrations of target compound and the competitor (see also below, determination of second-order rate constants). For the experiments with diselenides, the higher HOBr doses exceeded the concentration of the Se-compound. This is required because 3 moles of HOBr are consumed per mole of diselenide (see section: “Pathways for reactions between HOBr and the studied diselenides”). After 1 min reaction time, reaction solutions were diluted 1 : 100 to 10 mL amber crimp vials for DMSe and DMDSe quantification and transferred to HPLC vials for the quantification of DPSe, DPDSe, *N*-acetylated-SeMet, *N*-acetylated-SeCys_2_, resorcinol and TMB. DMSe, DMDSe, DPSe and DPDSe were immediately measured after experiments due to their volatility (DMSe and DMDSe) or observed instability in diluted aqueous solutions (DPSe and DPDSe). *N*-Acetylated-SeMet, *N*-acetylated-SeCys_2_ and resorcinol from experiments with DMSe and DMDSe were measured within one week and stored beforehand at 4 °C. The pH of reaction solutions was immediately measured after each experiment and did not deviate more than 0.05 units from the initial pH.

List of the selected organic Se compounds, including their structures, molecular masses, and the determined apparent second-order rate constants (pH 8) for their reactions with HOBr. Also shown are the published second-order rate constants for the reactions of HOBr with the analogous S compounds at pH 8. Resorcinol was used as competitor for all Se species except for DPDSe where 1,3,5-trimethoxybenze (TMB) was applied. Experimental conditions: pH 8, [PO_4_]_tot_ = 20 mM for DMSe, DMDSe, DPSe and acetylated SeMet; [PO_4_]_tot_ = 10 mM for DPDSe and acetylated SeCys_2_. For kinetic experiments with DMSe and DMDSe in seawater, the following experimental conditions were used: pH 8, [PO_4_]_tot_ = 20 mM, [NaCl] = 0.55 M, [KBr] = 840 μM. MeOH in the reaction vials (0.5–5%) did not impact the reaction of HOBr with the Se species and competitor, since a competition kinetics approach was applied. The ratios of the second-order rate constants for reactions of competitors and target compounds with HOBr are suboptimal for *N*-acetylated-SeMet and DMSe in seawater. The corresponding second-order rate constants for the target compounds are associated with some uncertainty (see footnote)

**Table:** 

Compound name	Compound structure	Molecular mass	*k*_app,pH 8,HOBr+Se_ [M^−1^ s^−1^]	Reactivity ratio (*k*_app,pH 8,HOBr+Se_ : *k*_app,pH 8,HOBr+competitor)_	*k*_app,pH 8,HOBr+S_ [M^−1^ s^−1^]
Experiments in buffered ultrapure water					
Dimethyl selenide (DMSe)	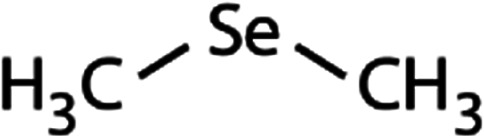	109	(7.1 ± 0.7) × 10^7^	3.9	(1.1 ± 0.2) × 10^9^^a^
Dimethyl-diselenide (DMDSe)	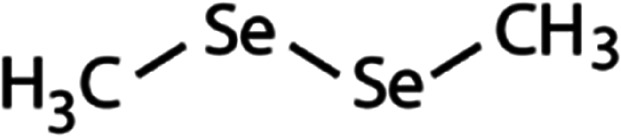	188	(4.3 ± 0.4) × 10^7^	2.3	Not known
Diphenyl selenide (DPSe)	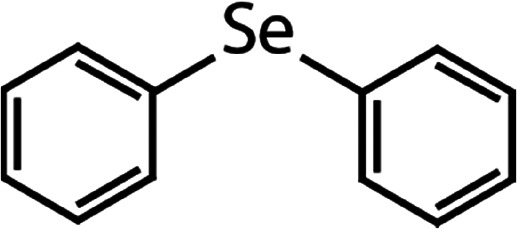	233	(3.5 ± 0.1) × 10^7^	1.9	Not known
Diphenyl diselenide (DPDSe)	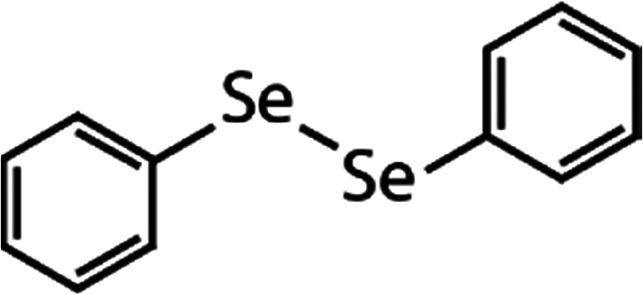	313	(8.0 ± 0.4) × 10^6^	2.8	Not known
*N*-Acetylated-selenomethionine (*N*-acetylated-SeMet)	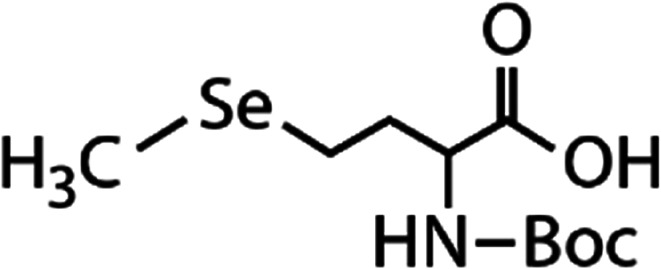	413.4	(2.8 ± 0.3) × 10^8^^c^	15.3^c^	(3.6 ± 0.3) × 10^6^^b^
*N*-Acetylated-selenocystine (*N*-acetylated-SeCys_2_)	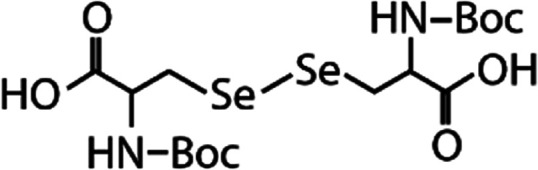	768.7	(3.8 ± 0.2) × 10^7^	2.1	(3.4 ± 0.8) × 10^5^^b^
Seawater experiments					
Dimethyl selenide (DMSe)	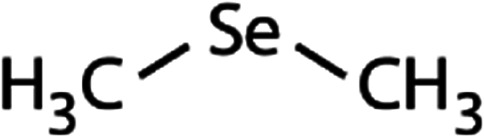	109	(4.7 ± 0.5) × 10^8^^c^	26.0	(1.2 ± 0.2) × 10^9^^a^
Dimethyl-diselenide (DMDSe)	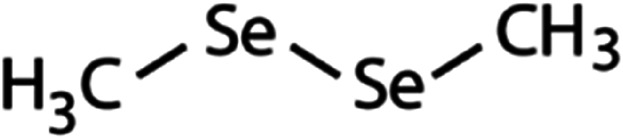	188	(4.5 ± 0.2) × 10^7^	2.5	Not known

**Table:** 

Second-order rate constants for the competitors					
Compound name	Compound structure	Molecular mass	*k*_HOBr+*X*_ [M^−1^ s^−1^]	Reactivity ratio (*k*_app,pH 8,HOBr+Se_ : *k*_app,pH 8,HOBr+competitor)_	*k*_app,pH 8,HOBr+*X*_ [M^−1^ s^−1^]
1,3,5-Trimethoxy-benzene (TMB)	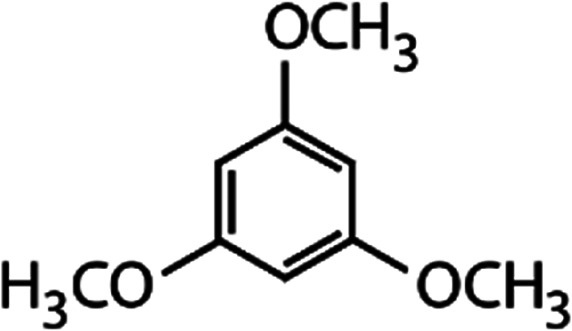	168.2	3.4 × 10^6^^d^	—	2.9 × 10^6^
Resorcinol	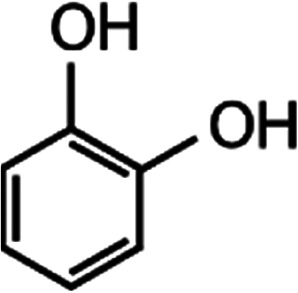	110.1	6.56 × 10^6^^e^	—	1.8 × 10^7^

a Ref. 24.

b Ref. 55, pH 7.2–7.5, *T* = 22 °C.

c Since the reactivity ratio between *k*_app,pH 8,HOBr+*N*-acetylated-SeMet_ and *k*_app,pH 8,HOBr+resorcinol_ is 15, the reaction conditions are suboptimal and the determined second-order rate constant of the reaction between HOBr and *N*-acetylated-SeMet is associated with some uncertainty. Likewise, the determined rate constant for the reaction between DMSe and HOBr in artificial seawater medium is subject to uncertainty since the reactivity ratio between *k*_app,pH 8,HOBr+DMSe_ and *k*_app,pH 8,HOBr+resorcinol_ is 26.

d Ref. 59.

e See Table S4.†

#### Analyses of organic Se compounds and the competitors

Concentrations of DMSe and DMDSe were quantified using direct-immersion solid-phase microextraction (DI-SPME) coupled to capillary gas chromatography-mass spectrometry (GC/MS) (DI-SPME-GC/MS) as described in Vriens *et al.* 2015.^37^ Concentrations of DPSe, DPDSe, *N*-acetylated-SeMet, *N*-acetylated-SeCys_2_, resorcinol and TMB were quantified by HPLC/UV, using a Dionex Ultimate 3000 HPLC system (Thermo Fisher Scientific, Waltham, Massachusetts, USA) and a Cosmosil C_18_ column (3.0 ID × 100 mm; Nacalai Tesque, Inc., Kyoto, Japan). For detailed information about the quantification of Se compounds and competitors, see Text S6, ESI.† The limits of quantification (LOQs) obtained for DMSe, DMDSe, DPSe, DPDSe, resorcinol and TMB were respectively, 11 ± 6 nM, 2.0 ± 1.2 nM, 96 ± 35 nM, 56 ± 9 nM, 182 ± 113 and 32 ± 9 nM (method for determination of LOQs described in Text S7, ESI†). LOQs for *N*-acetylated-SeMet and *N*-acetylated-SeCys_2_ were approximately 240 nM and 760 nM, respectively.

#### Determination of second-order rate constants

The second-order rate constants for the reactions between HOBr and the organic Se compounds were calculated based on the experimentally obtained slopes (plot of natural logarithm (ln) of the relative residual concentration of the Se compound *versus* the ln of the relative residual concentration of the competitor). Results of the individual kinetic experiments performed at pH 8, and the reported second-order rate constants of the reactions between HOBr and the competitors are provided in Tables S4–S6 (ESI†), respectively. Summary plots representing average slope values for all experiments are presented in Fig. S4 and S5, ESI.† They showed generally good linearity and reproducibility. Apart from the experiments using *N*-acetylated-SeMet and resorcinol and the experiment using DMSe and resorcinol in artificial seawater medium, the ratios of the second-order rate constants for the target compound and the competitor were <10, which indicates the feasibility of this approach (Tables S5 and S6, ESI†).

### Identification and (semi)quantification of Se-containing oxidation products

To investigate the products of the reactions of HOBr with selected organic Se compounds, batch experiments were performed in 10 mL amber gastight headspace vials using a 1 mM NaHCO_3_-buffer solution (pH 8) and variable molar HOBr : Se compound ratios (*i.e.*, 0 : 1 [Se compound blank], 1 : 1, 3 : 1 and 10 : 1). The initial concentration of the Se compounds was 6.25 μM. After 1 min of reaction time under magnetic stirring (*t*_1/2_ ≈ 0.01 seconds), the solutions were mixed with methanol containing 0.1% formic acid (volumetric 1 : 1 ratio) and analyzed by high resolution mass spectrometry (HR-MS) within the next 10 hours. The HR-MS system (QExactive HF Orbitrap™, Thermo Scientific, Switzerland) was equipped with an electrospray ionization source (ESI) and was calibrated before analyses (Pierce™ ESI solutions). All analyses were performed as direct infusion in the positive ionization mode. Selenium compounds in the recorded mass spectra were detected based on the specific Se isotopic pattern. The oxidation products were then identified using the software “Freestyle” (Thermo Scientific) and the Eawag tool “enviPath”.^38^ Once identified, a semi-quantification of the Se-containing oxidation products was carried out by recording single ion mode (SIM) spectra at their nominal mass for each condition, and the intensity was retrieved using the Freestyle software. Further details about HR-MS analyses are provided in the Text S9, ESI.† To quantify potential formation of inorganic Se (SeO_3_^2−^ [IV] and SeO_4_^2−^ [VI]), reaction products were additionally analyzed by liquid chromatography coupled to inductively coupled plasma tandem mass spectrometry (LC-ICP-MS/MS). The system consisted of an Agilent LC 1200 series coupled to an Agilent 8800 ICP-MS/MS. The chromatographic separation was adapted from a previous study,^39^*i.e.*, using an anion exchange column (Hamilton PRP-X100, 100 × 4.1 mm, 10 μm, with the appropriate guard column) and a gradient elution of ammonium citrate (4 to 10 mM; pH 5.2). The mobile phase was delivered at 1.25 mL min^−1^ and the injection volume was 25 μL.

## Results and discussion

### Reactivities of the selected organic selenium compounds with HOBr in buffered ultrapure water and artificial seawater

The apparent second-order rate constants (pH 8) for the reactions between the selected organic Se compounds and HOBr are provided in Table 1 and are in the range of (8.0 ± 0.4) × 10^6^ and (2.8 ± 0.3) × 10^8^ M^−1^ s^−1^, indicating a very high reactivity of all selected Se compounds. Monoselenides had significantly higher reactivities than the analogous diselenides with apparent second-order rate constants for DMSe, DPSe and *N*-acetylated-SeMet, respectively, 1.7, 4.4 and 7.4 times higher than for their diselenide analogs, *i.e.*, DMDSe, DPDSe, and *N*-acetylated-SeCys_2_ (Table 1). The relatively higher reactivity of monoselenides compared to diselenides can be explained by the lower oxidation state of Se in monoselenides (oxidation state of −II) as compared to diselenides (oxidation state of −I). Therefore, the electron density in Se in monoselenides is higher than for the corresponding diselenides, which makes monoselenides better nucleophiles. Apart from the effect of the Se oxidation state, we observed that Se compounds with alkyl substituents had a higher reactivity than those with aryl substituents. A reactivity enhancement by a factor of 2.1 and 5.3 was observed, respectively, for DMSe *versus* DPSe and DMDSe *versus* DPDSe (Table 1). Alkyl substituents have a positive inductive effect (*i.e.*, electron donating effect, +I effect), while aryl substituents have a negative resonance and a negative inductive effect (*i.e.*, electron withdrawing effect, −I effect).^40^ Therefore, alkyl substituents make the Se-atom more nucleophilic than aryl substituents, which is in line with the higher reactivities of alkylated compared to arylated Se species with the electrophilic HOBr.

A significantly higher reactivity (almost 1 order of magnitude) was observed for the DMSe–HOBr reaction in buffered artificial seawater (*k*_DMSe+HOBr,seaw._ = (4.7 ± 0.5) × 10^8^ M^−1^ s^−1^) than in ultrapure water (*k*_DMSe+HOBr_ = (7.1 ± 0.7) × 10^7^ M^−1^ s^−1^). It was tested if the ionic strength of seawater could explain the higher reactivity. Experiments were carried out in a perchlorate medium with the same ionic strength as the artificial seawater (but in absence of bromide (Br^−^) and chloride, to avoid alteration of the bromine speciation) (Table S6 and Fig. S5†).^41^ Based on these experiments it can be concluded that ionic strength effects as well as a higher Br^−^ concentration contribute to the higher reactivity (Text S10, ESI†). In contrast, no change in reactivity was observed for the reaction between DMDSe and HOBr when comparing ultrapure water with artificial seawater (Table 1). We currently do not have an explanation for the different behavior between DMSe and DMDSe in these tests. However, given the suboptimal conditions for the measurement of second-order rate constant for the reaction of HOBr with DMSe (large difference between target compound and competitor), this value should be considered with caution.

### Reactivity of SeMet with HOBr compared to other studies and oxidants

For the alkylated monoselenide compound *N*-acetylated-SeMet, an apparent second-order rate constant for the reaction with HOBr on the order of 3 × 10^8^ M^−1^ s^−1^ was obtained, which is higher than for DMSe. Because both compounds are selenoethers with two methyl groups at the Se-atom, this indicates that the Se atom in SeMet is the preferred site of HOBr attack at pH 8 and that the amino group only plays a minor role (*k*_app,pH 8,R-NH2+HOBr_ ≈ 10^6^ M^−1^ s^−1^).^41–43^ The apparent second-order rate constant determined here for the reaction between SeMet and HOBr is in the same order of magnitude as the values reported for the reactions between SeMet and SeCys with hypochlorous acid (HOCl) at pH 7.4, *i.e.*, (3.2 × 10^8^ M^−1^ s^−1^ and 8.5 ± 0.4 × 10^8^ M^−1^ s^−1^, respectively),^27,44^ however, it is 20 times higher than a previous value (1.4 × 10^7^ M^−1^ s^−1^) determined for the reaction between SeMet and HOBr.^44^ That value is also derived by competition kinetics (at a pH of 7.4), using *N*-acetyl-tyrosin (*N*-ac-Tyr) as a competitor. However, *N*-ac-Tyr has a much lower HOBr reactivity (*k*_HOBr+*N*-ac-Tyr_ = 2.6 × 10^5^ M^−1^ s^−1^) than SeMet, which is not ideal in experiments using competition kinetics (the ratio of the second-order rate constants is 50). In this study, resorcinol was applied, for which the reactivity is closer to HOBr–*N*-acetyl-SeMet reaction at the studied pH value (*k*_HOBr+resorcinol_ = 1.8 × 10^7^ M^−1^ s^−1^; pH 8, ratio of second-order rate constants is 15), and therefore the second-order rate constant determined in the current study is most likely closer to the expected value. Nevertheless, further experiments would be necessary to elucidate this discrepancy. To exclude a contribution of BOC_2_O to the observed reactivity (note that BOC_2_O was added in a 10-times molar excess relative to Se), we performed additional tests (Text S11, ESI†), indicating that the apparent second-order rate constant for the reaction of HOBr with BOC_2_O is about 10 M^−1^ s^−1^. Based on this observation, a contribution of the derivatization agent to the overall kinetics can be excluded. Further studies have investigated the reactions of organic selenium species with other oxidants than HOBr,^27^*i.e.*, with hypothiocyanous acid,^44^ chloramine,^45^ peroxynitrous acid^46^ and singlet oxygen.^27^ From these studied oxidants, the reactivity between HOBr and SeMet determined in the current study was the highest.

### Se-containing products from the reactions between organic selenium compounds and HOBr

#### Monoselenides

**Fig. 1:**
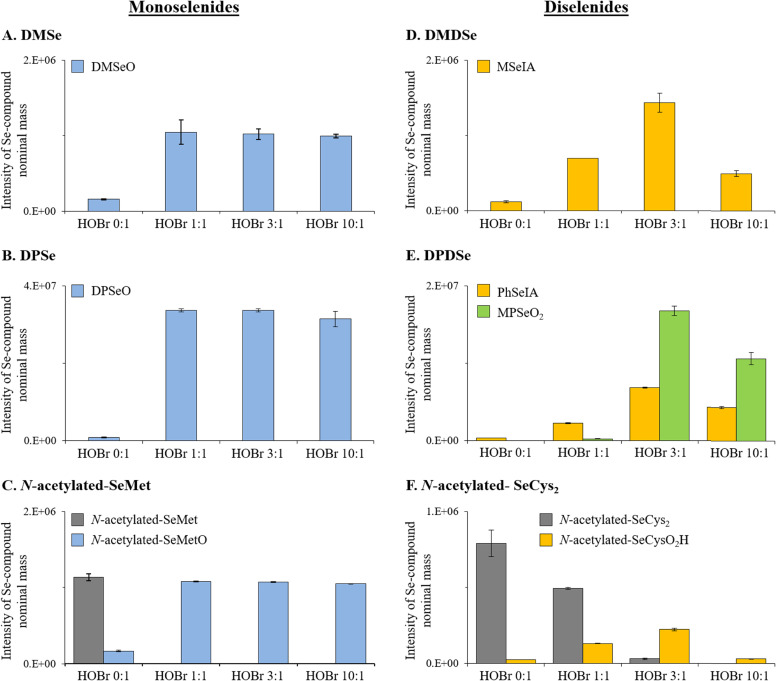
Semi-quantitative HR-MS data for the identified Se-containing products and the initial Se compounds when detected from the reactions between HOBr and (A) DMSe, (B) DPSe, (C) *N*-acetylated-SeMet, (D) DMDSe, (E) DPDSe and (F) *N*-acetylated-SeCys_2_. All reactions were performed with variable molar HOBr : Se compound ratios (*i.e.*, 0 : 1, 1 : 1, 3 : 1 and 10 : 1) at pH 8. Initial concentrations of organic Se compounds were 6.25 μM. The signal intensities are those of the Se compounds detected with a Na^+^ adduct. Data in all panels give the average value for triplicate analyses. For all data indicated, error bars present standard deviations.

For the reaction between DMSe and HOBr, dimethyl selenoxide was the only oxidation product identified by HR-MS (DMSeO; Table 2, Fig. S6, ESI†). It was confirmed by SPME-GC/MS analysis that DMSe was completely abated at a molar HOBr : DMSe ratio of 1 : 1 (data not shown). The HR-MS signal intensity for DMSeO indicated that its formation is complete at a molar HOBr : DMSe ratio of 1 : 1 and that even for HOBr : DMSe ratios up to 10 : 1, DMSeO is not further oxidized (Fig. 1A). Also, LC-ICP-MS/MS and LC-HRMS analyses indicated a single Se species for the experiment with a molar HOBr : DMSe ratio of 10 : 1 (Fig. S8–S10, ESI†) and neither selenite (Se[+IV]) nor selenate (Se[+VI]) were detected. Our results are consistent with previous studies using other oxidants (hydrogen peroxide [H_2_O_2_], ozone [O_3_] or hypochlorous acid [HOCl]), reporting DMSeO as main oxidation product.^35,47,48^ Higher oxidized products (*i.e.*, selenones such as dimethyl selenone [DMSeO_2_]) were only reported in organic solvents with oxidants such as HOCl, permanganate (MnO_4_^−^), and others.^47,49^

**Table:** Se-containing oxidation products of the reactions between HOBr and DMSe, DMDSe, *N*-acetylated-SeMet, DPSe, DPDSe, and *N*-acetylated-SeCys_2_ identified by HR-MS. Methane selenonic acid (MSeA) was not detected by HR-MS but suggested in analogy to the S-chemistry

Se-containing target compound	Se-containing oxidation products		
	Name	Chemical formula	Chemical structure
DMSe	Dimethyl selenoxide (DMSeO)	(CH_3_)_2_SeO	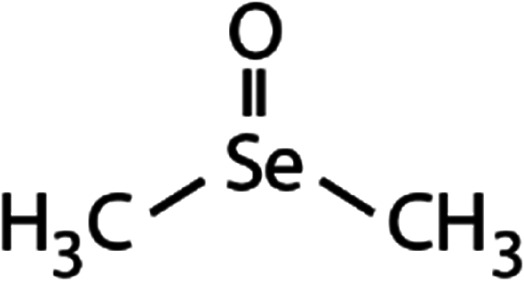
DPSe	Diphenyl selenoxide (DPSeO)	(C_6_H_5_)_2_SeO	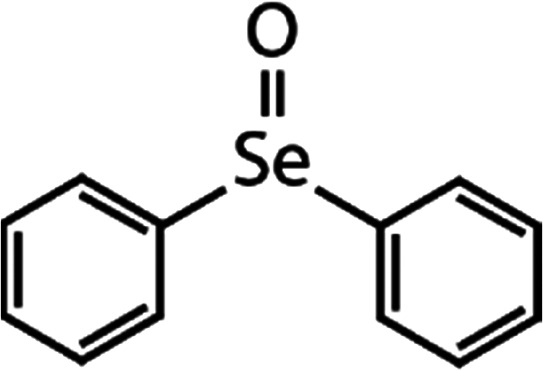
*N*-Acetylated-SeMet	*N*-Acetylated-selenomethionine-oxide (*N*-acetylated-SeMetO)	C_10_H_19_O_4_NSeO	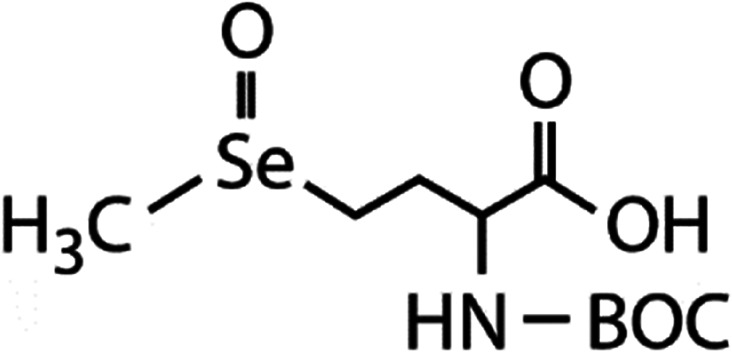
DMDSe	Methane seleninic acid (MSeIA)	CH_3_SeO_2_H	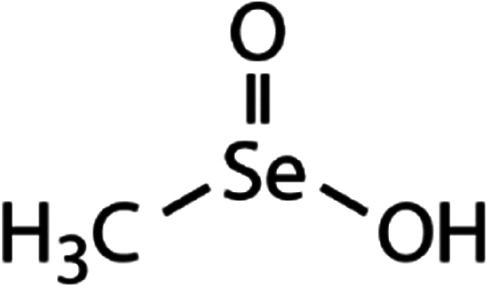
	Methane selenonic acid (MSeA)	CH_3_SeO_3_H	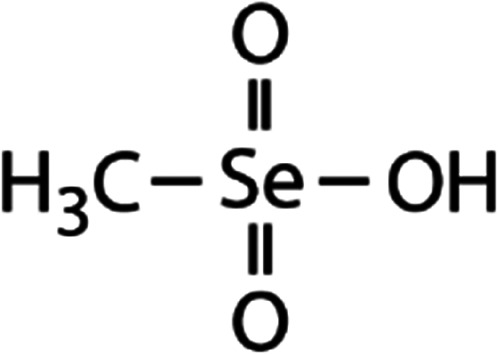
DPDSe	Phenyl seleninic acid (PhSeIA)	(C_6_H_5_)SeO_2_H	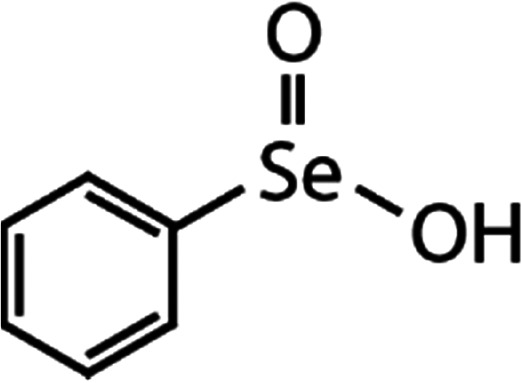
	Methyl phenyl selenone (MPSeO_2_)	C_7_H_8_SeO_2_	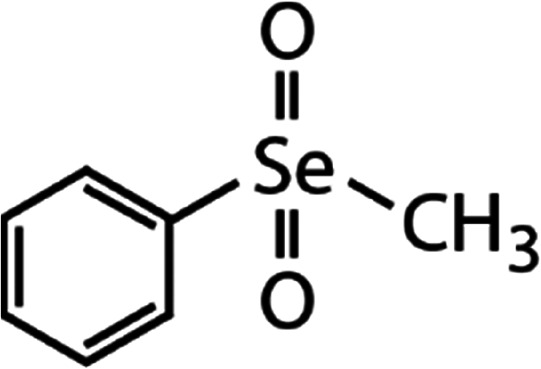
*N*-Acetylated-SeCys_2_	*N*-Acetylated-selenocysteine seleninic acid (*N*-acetylated-SeCysO_2_H)	C_8_H_14_O_4_NSeO_2_H	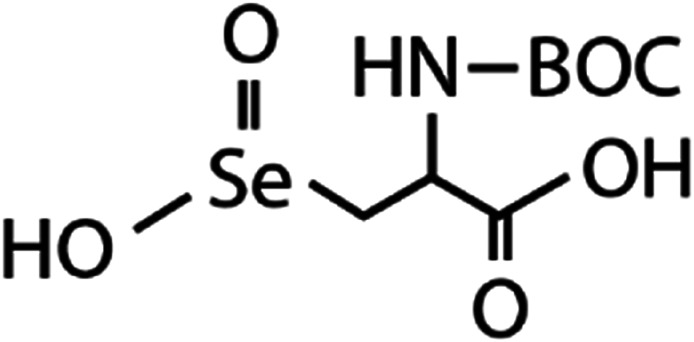

Similarly, for the other organic monoselenide compounds, *i.e.*, DPSe or *N*-acetylated-SeMet, only one oxidation product was detected, which were identified by HR-MS to be DPSeO and *N*-acetylated-SeMetO, respectively (Table 2, Fig. 1B and C, and S11 and S12, ESI†). HPLC/UV analyses confirmed that at a molar HOBr : Se compound ratio of 1 : 1, DPSe and *N*-acetylated-SeMet were completely consumed (data not shown). Also, the HR-MS signal intensities indicated that their formation was complete at a molar HOBr : Se compound ratio of 1 : 1 (Fig. 1B and C). Overall, these results indicate that the oxidation of monoselenides by HOBr proceeds *via* an oxygen transfer reaction with a 1 : 1 stoichiometry between HOBr and the target compounds as is known for organo sulfur halogenation, however, this was not specifically investigated in our study.

#### Diselenides

The reaction between DMDSe and HOBr yielded methane seleninic acid (MSeIA; Table 2), as was demonstrated both by HR-MS (Fig. S12, ESI†) and LC-ICP-MS/MS analyses (Fig. S13, ESI†). The formation of MSeIA increased when the molar HOBr : DMDSe ratio was increased from 1 : 1 to 3 : 1, and then its intensity decreased for a ratio of 10 : 1 (Fig. 1D and S14 and S15, ESI†). The decrease of MSeIA when increasing the molar HOBr : DMDSe ratio from 3 : 1 to 10 : 1 indicates a further reaction of MSeIA with HOBr. Stoichiometric experiments indicate the consumption of 3 moles HOBr per mole of DMDSe (Text S12, Fig. S22, ESI†). While a new Se peak could be observed by LC-ICP-MS/MS at a molar HOBr : DMDSe ratio of 10 : 1 (which did neither correspond to Se[+IV] nor Se[+VI]; Fig. S13, ESI†), no other Se-containing compound was detected by HR-MS, apart from DMSeO_2_ (Fig. S12, ESI†), which is derived from a DMSe impurity in the DMDSe standard (Fig. S23, ESI†).

DPDSe and *N*-acetylated-SeCys_2_ were oxidized by HOBr to phenyl seleninic acid (PhSeIA) and *N*-acetylated-selenocysteine-seleninic-acid (*N*-acetylated-SeCysO_2_H), respectively (Table 2, Fig. 1E and F and S19–S23, ESI†). For DPDSe, an additional product was detected, *i.e.*, methyl phenyl selenone (MPSeO_2_; Table 2 and Fig. S16, ESI†). PhSeIA, MPSeO_2_ and *N*-acetylated-SeCysO_2_H were already detected in blank samples of DPDSe and *N*-acetylated-SeCys_2_ (without HOBr addition) potentially due to their formation in the electrospray ionization. The concentrations of these compounds increased at molar HOBr : DPDSe or HOBr : *N*-acetylated-SeCys_2_ ratios of 1 : 1 and 3 : 1 (Fig. 1E and F), in line with the total consumption of DPDSe or *N*-acetylated-SeCys_2_ at a molar HOBr : Se compound ratio of 3 : 1 (HPLC/UV analyses, not shown). It is unclear why MPSeO_2_ is observed, but impurities of methylphenyldiselenide might be precursors. However, further investigations are needed to support this hypothesis. At molar HOBr : DPDSe or HOBr : *N*-acetylated-SeCys_2_ ratios of 10 : 1, PhSeIA, MPSeO_2_ and *N*-acetylated-SeCysO_2_, respectively, were present in lower concentrations than for the 3 : 1 ratios (Fig. 1E and F). A similar trend was also observed for MSeIA (Fig. 1D). This indicates that further products are formed, which could not be detected by HR-MS. Furthermore, neither Se(+IV) nor Se(+VI) were detected by LC-ICP-MS/MS for all investigated molar HOBr : DPDSe ratios. Only for the 3 : 1 and 10 : 1 molar HOBr : *N*-acetylated-SeCys_2_ ratios, a small proportion of Se(+IV) (*i.e.*, <10%) was tentatively detected (see Fig. S18, ESI†).

### Pathways for reactions between HOBr and the selected diselenides

Seleninic acids were the end products of reactions between diselenides and HOBr (Table 2, Fig. 1D and E). Our results indicate that 3 moles of HOBr per mole of diselenide were required for a complete abatement of these compounds (*i.e.*, DMDSe, DPDSe and *N*-acetylated-SeCys_2_) (Fig. S22, ESI†). Overall, the diselenide–HOBr reaction proceeds by the transfer of 6 electrons (eqn (1)–(3)). Thereby, 3 moles of HOBr (Br[+I]) are reduced to Br^−^ (Br[−I]) to oxidize 1 mole of diselenide (2× Se[−I]) to two moles of seleninic acid (Se[+II]), according to eqn (1)–(3).

1 Reduction reaction: 3HOBr + 6e^−^ + 3H^+^ → 3Br^−^ + 3H_2_O

2 Oxidation reaction: R–Se–Se–R + 4H_2_O → 2R–SeO–OH + 6e^−^ + 6H^+^

3 Redox: 3HOBr + R–Se–Se–R + H_2_O → 3Br^−^ + 2R–SeO–OH + 3H^+^

Since 3 moles of HOBr are consumed per mole of diselenide abated and per mole of seleninic acid produced (eqn (3)), it can be concluded that the second and third oxidation steps with HOBr are faster than the initial attack on the diselenides.

The formation of seleninic acid involves the rupture of the diselenide bond likely due to the weaker Se–Se than Se–C bond.^50^ Seleninic acid was also identified as the main product in other studies that investigated the oxidation of diselenides by H_2_O_2_ (ref. 35, 51 and 52) and O_3_.^48^ Two out of these four studies^35,48^ also reported a 1 : 3 stoichiometric ratio with a slower initial reaction step followed by faster reactions of the intermediates.

### Comparison of the bromine reactivities of selected Se compounds and the analogous S compounds

The apparent second-order rate constants for the reactions of organic Se compounds with HOBr can be compared to the analogous S compounds, *i.e.*, DMS, *N*-acetylated-Met and *N*-acetylated-Cys_2_. Previously, similar comparisons were made for the reaction between ^1^O_2_ and SeMet *vs.* Met, indicating around 10- to 4-fold^27,53^ higher reactivity of SeMet. The reactivity of SeMet with HOCl was about 10-fold higher than for Met.^54^ The apparent second-order rate constants for the reactions of *N*-acetylated-SeMet (2.8 ± 0.3 × 10^8^ M^−1^ s^−1^) and *N*-acetylated-SeCys_2_ (3.8 ± 0.2 × 10^7^ M^−1^ s^−1^) with HOBr obtained in the current study are around 2 orders of magnitude higher than the corresponding values of the analogous S-containing compounds *N*-acetylated-Met (3.6 × 10^6^ M^−1^ s^−1^)^55^ and *N*-acetylated-Cys_2_ (3.4 × 10^5^ M^−1^ s^−1^).^55^ The higher reactivity of organic Se compounds compared to the analog organic S compounds is often attributed to the higher nucleophilicity and polarizability of the Se atom compared to the S atom.^26,29,30,56^ In a computational study on H_2_O_2_ oxidation, investigating the influence of different hydrogen, alkyl and aryl substituents and type of chalcogens, the lower activation energy for diselenides than for disulfides was explained by the type of chalcogen (which acts as a nucleophile) having a larger effect on reactivity than the hydrogen, alkyl and aryl substituent.^57^ Also, our results for HOBr reactivity (Table 1), demonstrate a larger difference between Se- and S-species (about 2 orders of magnitude) than between Se compounds with alkyl- and aryl-substituents (about a factor of 2 to 5).

However, when comparing DMS and DMSe an opposite trend was observed. The apparent second-order rate constant of 1.2 × 10^9^ M^−1^ s^−1^ (ref. 24) for the reaction between DMS and HOBr exceeds the value for the reaction between DMSe and HOBr (*k* = 7.1 ± 0.7 × 10^7^ M^−1^ s^−1^) by more than one order of magnitude (Table 1). For the experiments carried out in a simplified seawater matrix, the reactivity difference is smaller but still about a factor of 3 (Table 1).

### Environmental implications

To assess if HOBr could play a role in the fate of DMSe in marine waters, in analogy to DMS,^24^ first-order rate constants for the DMSe–HOBr reaction were calculated and compared to the kinetics of photochemical oxidation (currently considered as the main DMSe sink). With an apparent second-order rate constant of the DMSe–HOBr reaction of (7.1 ± 0.7) × 10^7^ M^−1^ s^−1^ (4.7 ± 0.5 × 10^8^ M^−1^ s^−1^ for seawater) at pH 8 (Table 1) and an estimated HOBr steady-state concentration of ≈3 × 10^−14^ M^24^ the calculated first-order rate constant of the DMSe-HOBr reaction is ≈2.1 × 10^−6^ s^−1^ (1.4 × 10^−5^ s^−1^ in synthetic seawater). These values are 1–2 orders of magnitude lower than the reported first-order rate constants for DMSe photooxidation (surface degradation) of 2.1 × 10^−4^ s^−1^ for the open ocean.^58^ Therefore, in contrast to DMS, it seems that HOBr for the boundary conditions in this estimation does not play an important role in the fate of marine DMSe.

For the other studied species, which may occur naturally in seawater (*i.e.*, DMDSe, SeMet and SeCys_2_), the relevance of their reaction with HOBr depends on the factors driving the concentrations of these species as well as the presence of further oxidants, *e.g.*, ^1^O_2_ and H_2_O_2_ (ref. 27) and other processes such as UV-mediated degradation, which are to the best of our knowledge still not quantified.

Furthermore, it is unlikely that SeMet, SeCys_2_, DMSe and DMDSe affect HOBr concentrations in marine waters, since HOBr is controlled by iodide, DOM and DMS.^24,25^

## Conclusions

The determined apparent second-order rate constants (pH 8) for the reactions between HOBr and the organic Se compounds DMSe, DMDSe, SeMet (as *N*-acetylated-SeMet), and SeCys_2_ (as *N*-acetylated-SeCys_2_) as well as the industrially produced aryl compounds DPSe and DPDSe ranged between (8.0 ± 0.4) × 10^6^ and (2.8 ± 0.3) × 10^8^ M^−1^ s^−1^. Generally, monoselenides had a significantly higher reactivity than the analogous diselenides, explained by the lower oxidation state of Se of −II in monoselenides than in diselenides (−I). Furthermore, it was demonstrated that monoselenides reacted in a 1 : 1 stoichiometry (HOBr : Se) to selenoxides, while diselenides reacted in a 3 : 1 stoichiometry (HOBr : Se) to seleninic acids. For the reaction between DMSe and HOBr, dimethyl selenoxide was the only oxidation product observed, which is in agreement with studies that investigated the reaction of DMSe with other oxidants. Also, for the other organic monoselenide compounds, *i.e.*, DPSe or *N*-acetylated-SeMet, only one oxidation product was detected, *i.e.*, DPSeO and *N*-acetylated-SeMetO, respectively. For the diselenides DMDSe, DPDSe and *N*-acetylated-SeCys_2_ respectively, MSeIA, phenyl seleninic acid (PhSeIA) and *N*-acetylated-selenocysteine-seleninic-acid (*N*-acetylated-SeCysO_2_H) were detected as (main) oxidation products. HOBr is likely not a generally relevant sink for DMSe, in contrast to DMS. Due to a lack of data on concentrations of the other studied natural Se compounds in seawater and their further sinks, the relevance of HOBr for their fate can currently not be assessed.

## Conflicts of interest

The authors declare that they have no conflict of interest.

## Supplementary Material

EW-010-D3EW00787A-s001
