# Supplementary material for: Reactions of hypobromous acid with dimethyl selenide, dimethyl diselenide and other organic selenium compounds: kinetics and product formation
Source: Environ Sci (Camb). 2024 Jan 8;10(3):620–30. doi: 10.1039/d3ew00787a (PMC10905664; doi:10.1039/d3ew00787a)
Supplement: EW-010-D3EW00787A-s001 [file EW-010-D3EW00787A-s001.pdf]

Electronic Supplementary Material (ESI) for Environmental Science: Water Research & Technology.
This journal is © The Royal Society of Chemistry 2024

SUPPORTING INFORMATION

Reactions of bromine with organic selenium compounds: Kinetics and product formation

Emanuel Müller[†,#], Urs von Gunten[†,#, §], Julie Tolu[†,#], Sylvain Bouchet[†,#] and Lenny H.E. Winkel[†,#,*]

[†]Eawag, Swiss Federal Institute of Aquatic Science and Technology, Department of Water Resources and Drinking Water (W+T), Ueberlandstrasse 133, CH-8600 Duebendorf

[#]ETH Zurich, Swiss Federal Institute of Technology, Institute of Biogeochemistry and Pollutant Dynamics (IBP), Department of Environment Systems (D-USYS), Universitätsstrasse 16, 8092 Zürich

[§] School of Architecture, Civil and Environmental Engineering (ENAC), École Polytechnique Fédérale de Lausanne (EPFL), 1015, Lausanne, Switzerland

*Corresponding author:
Lenny H.E.Winkel
Email: lenny.winkel@eawag.ch,
phone: +41 58 765 5601

Number of pages: 39
Number of texts: 11
Number of tables: 9
Number of figures: 23

Table of Contents

Texts

Text S1: Suggested marine biotic pathways for Selenium and Se compounds **S5**

Text S2: Experimental procedures and list of chemicals **S5**

Text S3: Method for the production of N-acetylated-Selenomethionine and N-acetylated-Selenocystine **S8**

Text S4: Test for chloramine formation of N-acetylated-SeMet and N-acetylated-SeCys2 ....**S9**

Text S5: Quantification of Se organic compounds and competitors **S13**

Text S6: Determination of limits of quantification for organic selenium compounds and associated reaction competitors **S14**

Text S7: Calculation of second-order rate constants for the reactions of organic selenium compounds with HOBr **S15**

Text S8: Identification and (semi)quantification of Se-containing oxidation products by HR-MS **S19**

Text S9: Experiments investigating the higher reactivity of DMSe with HOBr in buffered artificial seawater medium compared to buffered ultrapure water **S33**

Text S10: Kinetics of the HOBr-BOC2O reaction **S25**

Text S11: Stoichiometry for reactions between diselenide compounds and HOBr **S36**

Tables

Table S1: Suggested marine biotic pathways for Se and the corresponding references **S5**

Table S2: Specifications of used chemicals **S6**

Table S3: Species-specific second-order rate constants for reactions between protonated/deprotonated HOBr and resorcinol species and pK_a values for different resorcinol species and HOBr **S11**

Table S4: Fractions of resorcinol-species (αRes, αRes-, αRes2-), HOBr/OBr- (αHOBr, αOBr-) and derived apparent second-order rate constants for the reactions between resorcinol species and HOBr as a function of the pH **S11**

Table S5: Slopes of competition kinetics experiments (reaction of the organic Se compound with HOBr in competition with the reaction of the competitor with HOBr) **S16**

Table S6: Slopes of competition kinetics experiments performed in buffered artificial seawater and perchlorate medium (= high ionic strength) medium **S17**

Table S7: Concentrations of halides, HOBr and H^+ in DMSe-HOBr experiments performed in buffered artificial seawater **S34**

Table S8: Concentrations and fractions of BrCl, Br_2O and Br_2 for used HOBr concentrations in buffered artificial seawater medium according to Table S8 at pH 8 ....**S34**

Table S9: Experimental conditions of the HOBr-BOC_2O kinetic experiment and the determined apparent second-order rate constants at pH 8 ………… **S36**

Figures

Figure S1: Total chlorine (i.e. free available chlorine (FAC = [HOCl] + [OCl^-]) and chloramines) as a function of added HOCl for (A) *N*-acetylated-SeMet (10.0 µM) and (B) *N*-acetylated-$SeCys_2$ (4.9 µM) ………… **S11**

Figure S2: Apparent second-order rate constant of the resorcinol-HOBr reaction as a function of pH (based on data "k_{app}" from Table S4) ………… **S13**

Figure S3: Eluent composition of the different HPLC/UV -methods for quantification of resorcinol, TMB, DPSe, DPDSe, *N*-acetylated-SeMet and *N*-acetylated-$SeCys_2$ ………… **S14**

Figure S4: Competition kinetics plots of the relative measured organic Se compound and competitor concentrations from kinetic experiments with HOBr performed in phosphate-buffered medium at pH 8 ………… **S18**

Figure S5: Competition kinetics plots of ln of the relative measured DMSe, DMDSe and competitor concentrations from kinetic experiments with HOBr performed in buffered artificial seawater medium/high ionic strength medium at pH 8 ………… **S19**

Figure S6: HR-MS mass spectra (between *m/z* 100 and 155) for solutions after the reaction between DMSe and HOBr ………… **S21**

Figure S7: LC-ICP-MS/MS chromatograms indicating ^{80}Se counts for solutions obtained after the reaction between DMSe and HOBr ………… **S22**

Figure S8: Semi-quantitative HR-MS data for the product of the reaction between DMSe and HOBr, i.e., DMSeO ………… **S23**

Figure S9: Semi-quantitative LC-ICP-MS/MS data for DMSeO, confirming the HR-MS data in Figure S8 ………… **S23**

Figure S10: HR-MS mass spectra (between *m/z* 200 and 280) for solutions after the reaction between DPSe and HOBr (two molar ratios) ………… **S24**

Figure S11: HR-MS mass spectra (between *m/z* 300 and 365) for solutions after the reaction of *N*-acetylated-SeMet and HOBr (two molar ratios) ………… **S25**

Figure S12: HR-MS mass spectra (between *m/z* 100 and 170) for solutions after the reaction between DMDSe and HOBr (two molar ratios) ………… **S26**

Figure S13: LC-ICP-MS/MS chromatograms obtained for solutions after the reaction between DMDSe and HOBr (four molar ratios) ………… **S27**

Figure S14: Semi-quantitative HR-MS data for the reaction between DMDSe and HOBr (four molar ratios) ………… **S28**

Figure S15: Semi-quantitative LC-ICP-MS/MS data for the reaction between DMDSe and HOBr (four molar ratios) ………… **S28**

Figure S16: HR-MS mass spectra for solutions after the reaction between DPDSe and HOBr (two molar ratios) …… **S29**

Figure S17: HR-MS mass spectra for solutions after the reaction between *N*-acetylated--$SeCys_2$ and HOBr (three molar ratios) …… **S30**

Figure S18: LC-ICP-MS/MS chromatograms indicating ^{80}Se counts for solutions obtained after the reaction between *N*-acetylated-$SeCys_2$ (in the figure displayed as $SeCys_2$) and HOBr for four molar ratios …… **S31**

Figure S19: Semi-quantitative HR-MS data for the product of the reaction between *N*-acetylated-$SeCys_2$ (in the figure displayed as $SeCys_2$) and HOBr (four molar ratios) …… **S32**

Figure S20: Semi-quantitative LC-ICP-MS/MS data for the reaction between *N*-acetylated-$SeCys_2$ (in the figure displayed as $SeCys_2$) and HOBr (four molar ratios) …… **S32**

Figure S21: Kinetics of the HOBr- BOC_2O reaction (pseudo-first order conditions, see Table S9) at pH 8 …… **S36**

Figure S22: Stoichiometries of reactions between diselenides and HOBr at pH 8 (n=1). (A) DMDSe, (B) DPDSe, (C) *N*-acetylated-$SeCys_2$ …… **S37**

Figure S23: Measured DMS in DMDSe samples via DI-SPME-GC/MS …… **S37**

References …… **S38**

Text S1: Suggested marine biotic pathways for Selenium and Se compounds

Table S1: Suggested marine biotic pathways for Selenium and Se compounds

Pathway number	Description of pathway / Observation reported	Reference(s)
1	SeIV/SeVI uptake by algae	Obata et al. 2004[1]
2	DMSeP production in microalgae	Larsen et al. 2001[2]
3	Microalgae DMSe production, likely from selenonium derivative such as MeSeMet or DMSeP	Fan et al. 1997[3]
4	Intracellular presence of methylated selenocysteine in microalgae	Gómez-Jacinto et al. 2012[4]<br>Wrench 1978[5]<br>Bottino et al. 1984[6]
5	Release of organic Se from algae cells	Hu et al. 1997[7]
6	Abiotic degradation of $SeCys_2$ and SeMet in marine waters	Amouroux et al. 2000[8]
7	DMSePd (org Se) uptake by bacteria	Keine et al. 1998[9]
8	Inorganic Se uptake by marine bacteria	Brock et al. 2013[10]<br>Van Fleet-Stalder et al. [11]
9	DMSeP production by marine bacteria (hypothesized)	Brock et al. 2013[10]
10	DMSeP cleavage to DMSe in marine bacteria	Ansede & Yoch 1997[12]<br>Dickschat et al. 2010[13]
11	DMSeP demethylation to MeSeH in marine bacteria	Dickschat et al. 2010[13]
12	Production of DMDSe from MeSeH	Gabel-Jensen et al. 2010[14]

Text S2: Experimental procedures and list of chemicals

All glassware was cleaned with 0.5 % nitric acid (Merck, Hohenbrunn, Germany) for 24 hours, rinsed with ultrapure water (18 MΩ; Thermo Fisher, Nanopure, Reinach, Switzerland) and then methanol (CH_3OH, LC/MS grade, Fisher Scientific, Loughborough, UK), and finally dried under a fume hood before use. All dilutions and compound transfer steps were performed with gastight syringes (Hamilton, 1700 series, Bonaduz, Switzerland).

Table S2: Specifications of used chemicals

Chemical	Abbreviation	CAS number	Supplier	Purity	Usage	Stock solutions	Storage conditions (for the highest concentrated solution)
Acetonitrile	ACN, C_2H_3N	75-05-8	Fisher Chemical	LC/MS grade	HPLC/UV eluent	-	at 20 °C, in the dark
2,2′-Azino-bis(3-ethylbenzothiazoline-6-sulfonic acid) diammonium salt	ABTS	30931-67-0	Sigma-Aldrich	>98%	For quenching HOBr in the BOC_2O kinetic test. λ=405 nm; ε=30226 M^{-1} cm^{-1}	1 g/L in H_2O	at 4°C, replacement every week
Deuterated dimethyl sulfide	DMS-d_6, $(CD_3)_2$	926-09-0	Sigma-Aldrich	99%	Internal standard for DMSe	≈10^{-2} M and ≈10^{-5} M	at -18°C
N,N-Diethyl-*p*-phenylenediamine sulfate salt	DPD	6283-63-2	Sigma-Aldrich	>98%	used for chloramine test assay	For production of an acidified DPD solution	at 20 °C in the dark
Dimethyl diselenide	DMDSe, $(CH_3Se)_2$	7101-31-7	Sigma-Aldrich	96%	For kinetic experiments and product analysis	25 mM in methanol	at -18°C
Dimethyl selenide	DMSe, $(CH_3)_2Se$	593-79-3	Sigma-Aldrich	99%	For kinetic experiments and product analysis	25 mM in methanol	at -18°C
Diphenyl diselenide	DPDSe, $(C_6H_5Se)_2$	1666-13-3	Sigma-Aldrich	98%	For kinetic experiments and product analysis	6 mM in methanol	at 20 °C in the dark
Diphenyl selenide	DPSe, $(C_6H_5)_2Se$	1132-39-4	Sigma-Aldrich	96%	For kinetic experiments and product analysis	25 mM in methanol	at 4°C
Di-sodium hydrogen phosphate dihydrate	$Na_2HPO_4 \cdot 2H_2O$	10028-24-7	Sigma-Aldrich	≥99%	Buffer for kinetic experiments; used for chloramine test assay	40 mM for kinetic experiments, 0.4 M for chloramine test assay	at 4°C
Di-tert-butyl dicarbonate	BOC_2O, $C_{10}H_{18}O_5$	24424-99-5	Sigma-Aldrich	≥99%	Prod. of *N*-acetylated-$SeCys_2$	390 mM in methanol	at 4°C
Ethylenediamine tetraacetate disodium salt dihydrate	EDTA-Na_2	6381-92-6	Sigma-Aldrich	99-101% (titration)	used for chloramine test assay	Prod. of a 0.4 M PO_4 buffer	at 4°C
Formic acid	FA, CH_2O_2	64-18-6	Sigma-Aldrich	>98%	For Se-product analysis by HR-MS	10%	at 20 °C, in the dark

Hypobromous acid*	HOBr		own production		Oxidant for kinetic experiments	10^{-1} M in H_2O. Produced via the HOCl-Br^- reaction.	at 4°C, replacement every 2 weeks.
Mercury chloride	$HgCl_2$	7487-94-7	Sigma-Aldrich	≥99.5%	used for chloramine test assay	Prod. of a 0.4 M PO_4 buffer	at 4°C
Methane seleninic acid	MSeIA, CH_3SeO_2H	28274-57-9	Sigma-Aldrich	95%	For Se-product analysis by LC-ICP-MS/MS	12.7 mM	at 4°C
Methanol	MeOH, CH_3OH	67-56-1	Fischer Scientific	LC-MS grade	For cleaning, stock solutions, HR-MS analysis		at 20 °C, in the dark
Nitric acid	HNO_3	7697-37-2	Merck	ACS reagent grade , assay ≥65%	For cleaning vials; acidification of HOBr	10%	at 20 °C, in the dark
Phosphoric acid	H_3PO_4	7664-38-2	Merck	85%	HPLC/UV eluent	10 mM	at 20 °C, in the dark
Potassium bromide	KBr	7758-02-3	Sigma-Aldrich	>99%	HOBr prod.; for ASW	840 µM	at 4°C
Potassium dihydrogen phosphate	KH_2PO_4	7778-77-0	Fluka	≥99.5%	used for chloramine test assay	prod. of a 0.4 M PO_4 buffer for chloramine test assay	at 4°C
Potassium perchlorate	$KClO_4$	7778-74-7	Sigma-Aldrich	≥99%	Salt to adjust ionic strength	125 mM	at 20 °C
Resorcinol	Res, Benzene-1,3-diol, $C_6H_4(OH)_2$	108-46-3	Sigma-Aldrich	≥99%	For kinetic experiments (competitor)	250 mM in H_2O	at 4°C
Seleno-L-cystine	SeCys2, $C_6H_{12}N_2O_4Se_2$	2897-21-4	Sigma-Aldrich	>95%	Prod. of *N*-acetylated-SeCys2	0.6 mM in H_2O	at 4°C
Seleno-DL-methionine	SeMet, $C_5H_{11}NO_2Se$	1464-42-2	Sigma-Aldrich	≥99%	Prod. of *N*-acetylated-SeMet	6 mM in H_2O	at 4°C
Sodium chloride (recrystallized)	NaCl	7647-14-5	Merck	>99.5%	For artificial seawater production	0.55 M	at 4°C
Sodium hydrogen carbonate	$NaHCO_3$	144-55-8	Merck	Reagent grade, 99.0-101.0% (acidimetric)	For acetylation of SeMet and SeCys2; buffer for HR-MS analysis	1 mM for the use as a buffer	at 20 °C
Sodium hypochlorite	NaOCl	7681-52-9	Sigma-Aldrich	6-14% active chlorine	Prod. of HOBr; used in the chloramine test assay	(OCl^-: λ = 292 nM, ε = 350 M^{-1} cm^{-1}). 1 mM for the chloramine test assay	at 4°C

Selenate	SeO_4^{2-}	13410-01-0	Spec-tracer	ICP mass spectrometry standard	For Se-product analysis by LC-ICP-MS/MS	12.66 mM	at 4°C
Selenite	SeO_3^{2-}	10102-18-8	Spec-tracer	ICP mass spectrometry standard	For Se-product analysis by LC-ICP-MS/MS	12.66 mM	at 4°C
Sulfuric acid	H_2SO_4	7664-93-9	Sigma-Aldrich	ACS reagent grade (95-98%)	used for chloramine test assay	2%, for production of an acidified DPD solution	20 °C, in the dark
1,3,5-Trimethoxybenzene	TMB, $C_6H_3(OCH_3)_3$	621-23-8	Sigma-Aldrich	≥99%	For kinetic experiments (competitor)	2.5 mM in H_2O	at 4°C

Further information:

*As HOBr is not stable, its concentration was determined before each experiment spectrophotometrically (via OBr^-): λ=329 nm, ε=345 M^{-1} cm^{-1}.[15]

ASW: artificial seawater medium: [NaCl] = 0.55 M; [KBr] = 840 µM

prod. = production

Indicated temperatures of -18 °C, 4 °C and 20 °C indicate storage in the freezer, refrigerator and at room temperature, respectively.

Text S3: Method for the production of *N*-acetylated-Selenomethionine and *N*-acetylated-Selenocystine

The method used to produce *N*-acetylated-selenomethionine (*N*-acetylated-SeMet) and *N*-acetylated-selenocystine (*N*-acetylated-$SeCys_2$) is described in *McCurry et al. 2016*. [16] Here, we used 20 mL amber glass vials with screw caps. A solution of 390 mM BOC_2O in methanol was produced in a 10 mL headspace amber crimp vial and stored in the refrigerator at 4 °C. 5 mL methanol (CH_3OH, LC/MS grade, Fisher Scientific, Loughborough, UK) was added to each vial, followed by the addition of BOC_2O and the Se-amino acid to a total volume of 6 mL and a molar BOC_2O:Se ratio of 10:1. Finally, 250 g sodium hydrogen carbonate ($NaHCO_3$, analytical grade, Merck, Hohenbrunn, Germany) was added to the vial. The vial was then placed in a beaker half-filled with ultrapure water and sonicated for 30 min in an ultrasonic bath (Sonorex Super 10 P, Bandelin electronic GmbH & Co., Berlin, Germany). The cap of the vial was slightly opened to avoid overpressure due to CO_2 formation. After sonication and settling of solid $NaHCO_3$, 5 mL of the sonicated *N*-acetylated-SeMet solution was transferred to a 20 mL amber glass vial and mixed with 15 mL H_2O. The sonicated *N*-acetylated-$SeCys_2$ solution was directly filtered

(without dilution with water) through a 0.45 µm cellulose nitrate syringe filter (Whatman, Luer connection) and transferred to a 8 mL amber glass vial.

Stock solutions of *N*-acetylated-SeMet and *N*-acetylated-$SeCys_2$ were then quantified for total Se by ICP-MS/MS and the results were in good agreement with the calculated target concentrations (<3% deviation). In a separate experiment, the yield of *N*-acetylation of amines via BOC_2O was tested by formation of chloramines (Text S4).

Text S4: Test for chloramine formation of *N*-acetylated-SeMet and *N*-acetylated-$SeCys_2$

Chlorination of *N*-acetylated Se amino acids was performed to examine the effectiveness of the derivatization. For fully derivatized amino acids, the *N*-acetylated amino group can no longer react with HOCl, wherefore, the added chlorine remains in solution and can be detected photometrically by the *N,N*-diethyl-*p*-phenylenediamine (DPD) method. [17] If the amino acids are not or only partially derivatized, chlorine reacts with the amino group to the corresponding chloramines and can no longer be measured directly by DPD. However, upon addition of iodide, hypoiodous acid is formed, which reacts with DPD. [18] Experiments were carried out with *N*-acetylated-SeMet and various doses of HOCl in 10 mL amber glass vials under constant stirring. Water and *N*-acetylated-SeMet (final concentration = 10 µM) were added to the vial. The reaction was initiated by the addition of HOCl (final concentration: 0-50 µM; total volume = 4 mL). After one minute reaction time, 2.5 mL of the reaction solution was transferred to a 1 cm quartz cuvette (Helma Analytics, Müllheim, Germany), where 125 µL of a 0.4 M PO_4-buffer solution (containing 269 µM EDTA and 74 µM $HgCl_2$) and 125 µL of a DPD solution (containing 4.2 mM *N,N*-diethyl-p-phenylenediamine sulfate salt, 537 µM EDTA, 2% H_2SO_4) were previously added. EDTA and $HgCl_2$ serve to complex metals and iodide, respectively and thereby prevent interferences and potential formation of HOI (from the reaction between chloramines and iodide). The absorption signal was measured 15 seconds later (to ensure the required reaction time and a constant absorption signal) with a Cary Bio 100 UV-vis spectrophotometer (Varian, Palo Alto, California, USA) at λ = 510 nm. Afterwards, a few crystals of potassium iodide (KI, Merck, ≥99.5%) were

added to the cuvette and dissolved, thereby ensuring that [KI] > [Hg^{2+}]. The absorption was then measured again and the obtained signal was compared with the first reading. A higher second reading indicates chloramine formation and therefore an incomplete acetylation of the amine group. An unaltered signal indicates the absence of chloramines and thus unreactive amine groups, demonstrating that the *N*-acetylation procedure was effective.

Tests for *N*-acetylated-$SeCys_2$ were performed similarly to the procedure described for SeMet but using a reaction volume of 3.5 mL and a final *N*-acetylated-$SeCys_2$ concentration of 4.9 µM.

No chloramine formation was observed in the experiment with *N*-acetylated-SeMet, as the signal before and after KI-addition is identical (Figure S1A). It is also visible that the difference between added HOCl and quantified total chlorine (via oxidized DPD) corresponds exactly to the concentration of *N*-acetylated-SeMet (with a slope of 1 regarding Δtotal chlorine/Δadded HOCl after the reaction of HOCl with Se in the *N*-acetylated-SeMet). However, for *N*-acetylated-$SeCys_2$ chloramine formation is observed which indicates an incomplete *N*-acetylation of the amino group (Figure S1B). Ca. 20 µM HOCl are consumed by *N*-acetylated-$SeCys_2$. HOCl consumption can be explained by the three-step-oxidation of diselenides as seen for HOBr (see main text) and oxidation of non-*N*-acetylated amino groups. Quantified total chlorine after KI-addition is up to 3 µM (average 1.6 µM) higher compared to the first reading. However, the concentration of produced chloramine for different HOCl doses (representing the different data points in Figure S1) is inconsistent and the fraction of incomplete *N*-acetylated amino groups is difficult to predict (2.5 – 58%, average: 33%). Despite the still available amino groups in solutions of *N*-acetylated-$SeCys_2$, it can be excluded that the amino group will influence the kinetics of the reaction between *N*-acetylated-$SeCys_2$ and HOBr, because (i) the reactivity between HOBr and primary amines is around 10^6 M^{-1} s^{-1} at pH 8 [19, 20] which is 1-2 orders of magnitude lower than the observed reactivity between HOBr and *N*-acetylated-$SeCys_2$ and (ii) there is no HOBr-reactivity difference reported for methionine and *N*-acetylated methionine, [19] which points to a limited influence of the amino group for the overall reactivity.

The apparent second-order rate constants for the reactions between resorcinol and HOBr/OBr^- can be expressed by equation S1, with values indicated in Table S4 and graphic representation in Figure S2:

$$k_{app}(\text{Res+HOBr}) = k(\text{Res+HOBr}) \times \alpha\text{Res} \times \alpha\text{HOBr} + k(\text{Res-+HOBr}) \times \alpha\text{Res}^{-} \times \alpha\text{HOBr} + k(\text{Res}^{2-}\text{+HOBr}) \times \alpha\text{Res}^{2-} \times \alpha\text{HOBr} + k(\text{Res}^{2-}\text{+OBr}^{-}) \times \alpha\text{Res}^{2-} \times \alpha\text{OBr}^{-} \qquad \text{(equation S1)}$$

where

- k_{app}(Res+HOBr) is the pH-dependent apparent second-order rate constant of the HOBr-resorcinol reaction
- k(Res+HOBr), k(Res^-+HOBr), k(Res^{2-}+HOBr) and k(Res^{2-}+OBr^-) are the species-specific second-order rate constants for the reactions between protonated/deprotonated resorcinol species and HOBr (as indicated in Table S3)
- αRes, αRes^- and αRes^{2-} are the fractions of protonated and deprotonated resorcinol-species based on pK_a values (Table S4) and the actual pH
- αHOBr, αOBr^- the protonated and deprotonated fractions of HOBr/OBr^- based on pK_a values (Table S4) and the actual pH

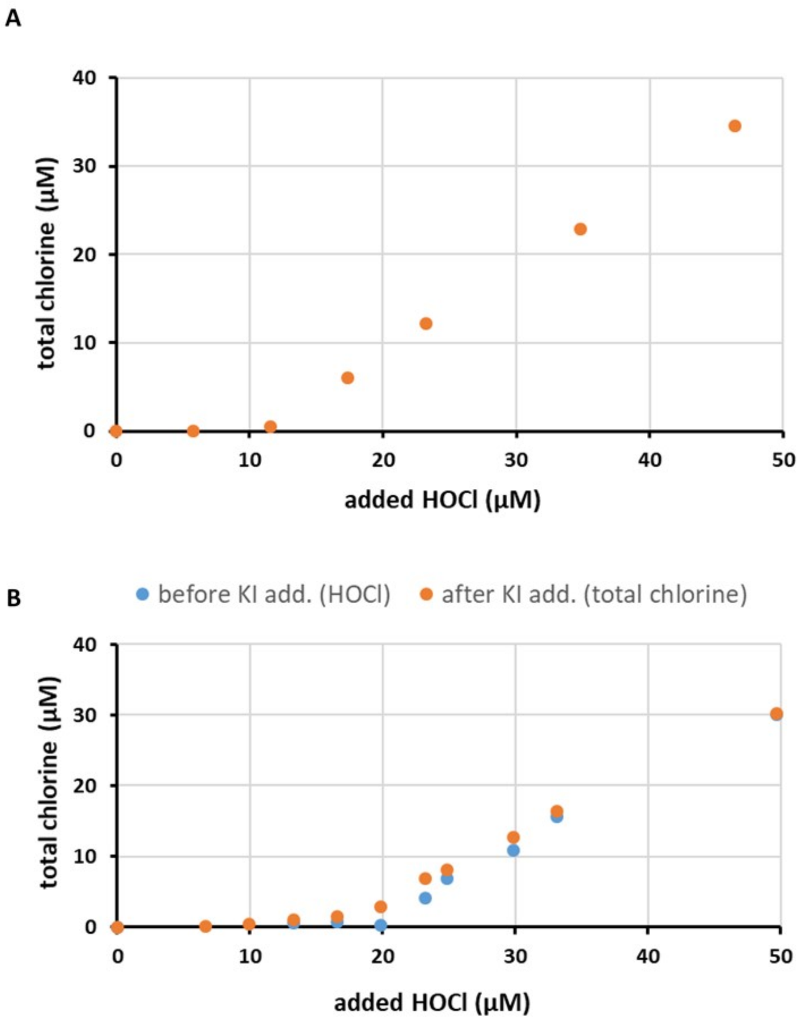

Figure S1: Total chlorine (i.e. free available chlorine (FAC = [HOCl] + [OCl^-]) and chloramines) as a function of added HOCl for (A) *N*-acetylated-SeMet (10.0 µM) and (B) *N*-acetylated-$SeCys_2$ (4.9 µM). FAC and total chlorine were quantified photometrically via oxidation of DPD at λ = 510 nm before and after KI addition, respectively. For Panel A, only values are shown after KI addition because of an insignificant difference to values before KI addition.

Table S3: Species-specific second-order rate constants for reactions between protonated/deprotonated HOBr and resorcinol species and pK_a values for different resorcinol species and HOBr.

**Reaction or acid-base equilibrium**	**Species-specific second-order rate constants or pKa values**	**Unit**	**Reference**
k(Res+HOBr)	7.90×10^6	$M^{-1}\ s^{-1}$	Criquet et al. 2015[21]
k(Res^-+HOBr)	3.50×10^8	$M^{-1}\ s^{-1}$	Criquet et al. 2015[21]
k(Res^{2-}+HOBr)	2.10×10^9	$M^{-1}\ s^{-1}$	Criquet et al. 2015[21]
k(Res^{2-}+OBr^-)	1.50×10^6	$M^{-1}\ s^{-1}$	Criquet et al. 2015[21]
pK_a(Res/Res^-)	9.4		Criquet et al. 2015[21]
pK_a(Res^-/Res^{2-})	11.2		Criquet et al. 2015[21]
pK_a(HOBr/OBr^-)	8.8		Troy & Margerum 1991[22]

Table S4: Fractions of resorcinol-species (αRes, αRes-, αRes2-), HOBr/OBr- (αHOBr, αOBr-) and derived apparent second-order rate constants for the reactions between resorcinol species and HOBr as a function of the pH: (*k*(Res+HOBr), *k*(Res^-+HOBr), *k*(Res^{2-}+HOBr), *k*(Res^{2-}+OBr^-)). The sum of *k*(Res+HOBr), *k*(Res^-+HOBr), *k*(Res^{2-}+HOBr) and *k*(Res^{2-}+OBr^-) displays the apparent second-order rate constant of the resorcinol-HOBr reaction (k_{app}) at a given pH.

**pH**	**αRes**	**αRes^-**	**αRes^{2-}**	**αHOBr**	**αOBr^-**	***k*(Res+HOBr)**	***k*(Res^-+HOBr)**	***k*(Res^{2-}+HOBr)**	***k*(Res^{2-}+OBr^-)**	**k_{app}**
						$M^{-1}\ s^{-1}$	$M^{-1}\ s^{-1}$	$M^{-1}\ s^{-1}$	$M^{-1}\ s^{-1}$	$M^{-1}\ s^{-1}$
6	0.9996	0.0004	0.0000	0.9984	0.0016	7.88×10^6	1.39×10^5	5.26×10^0	5.96×10^{-6}	**8.02×10^6**
7	0.9960	0.0040	0.0000	0.9844	0.0156	7.75×10^6	1.37×10^6	5.17×10^2	5.85×10^{-3}	**9.11×10^6**
8	0.9617	0.0383	0.0000	0.8632	0.1368	6.56×10^6	1.16×10^7	4.38×10^4	4.95×10^0	**1.82×10^7**
9	0.7153	0.2847	0.0018	0.3869	0.6131	2.19×10^6	3.86×10^7	1.45×10^6	1.64×10^3	**4.22×10^7**
10	0.2008	0.7992	0.0474	0.0594	0.9406	9.41×10^4	1.66×10^7	5.91×10^6	6.69×10^4	**2.27×10^7**
11	0.0150	0.6131	0.3869	0.0063	0.9937	7.44×10^2	1.35×10^6	5.09×10^6	5.77×10^5	**7.02×10^6**
12	0.0003	0.1368	0.8632	0.0006	0.9994	1.71×10^0	3.02×10^4	1.14×10^6	1.29×10^6	**2.47×10^6**

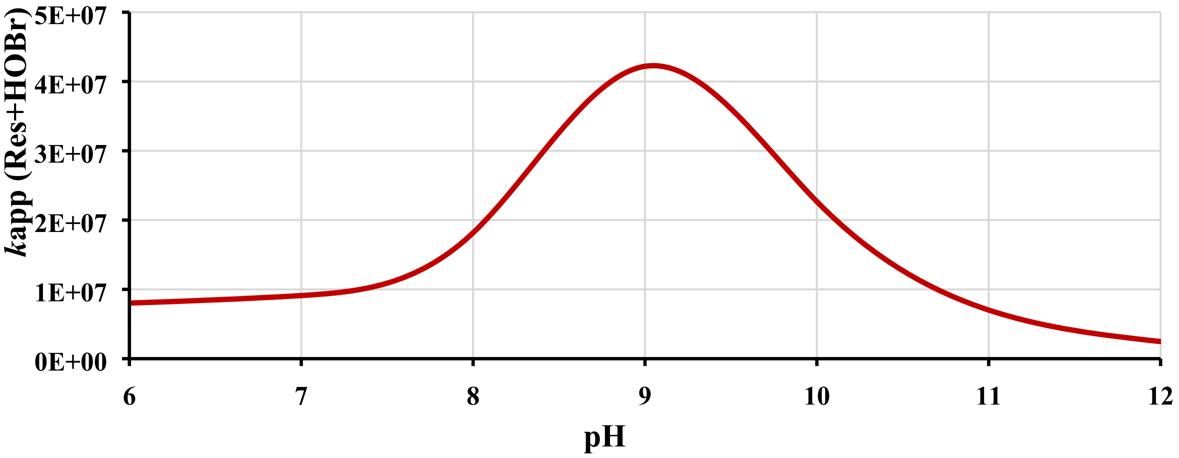

Figure S2: Apparent second-order rate constant of the resorcinol-HOBr reaction as a function of pH (based on data "k_{app}" from Table S4)

Text S5: Quantification of Se organic compounds and competitors

We used deuterated dimethyl sulfide (DMS-d6) as an internal standard for DMSe, as described in *Vriens et al. 2015*. [23] For DMDSe, better results were obtained when its quantification was based on counts (without using an internal standard). Concentrations of DPSe, DPDSe, *N*-acetylated-SeMet, *N*-acetylated-$SeCys_2$, resorcinol and TMB were quantified by HPLC/UV, using a Dionex Ultimate 3000 HPLC system (Thermo Fisher Scientific, Waltham, Massachusetts, USA) and a Cosmosil C_{18} column (3.0 ID X 100 mm; Nacalai Tesque, Inc., Kyoto, Japan). Separation of DPSe and DPDSe was achieved with an isocratic elution using a methanol/10 mM phosphoric acid eluent, whereas *N*-acetylated-SeMet, *N*-acetylated-$SeCys_2$ and resorcinol were separated using a gradient elution, with an acetonitrile/10 mM phosphoric acid eluent (Figure S3). For TMB quantification, an acetonitrile/10 mM phosphoric acid eluent was used under isocratic conditions (Figure S3). A photodiode array detector (Dionex PDA-3000) was used to quantify DPSe, DPDSe, *N*-acetylated-SeMet, *N*-acetylated-$SeCys_2$, resorcinol and TMB at 254 nm, 254 nm, 220 nm, 208 nm, 273 nm, and 208 nm, respectively.

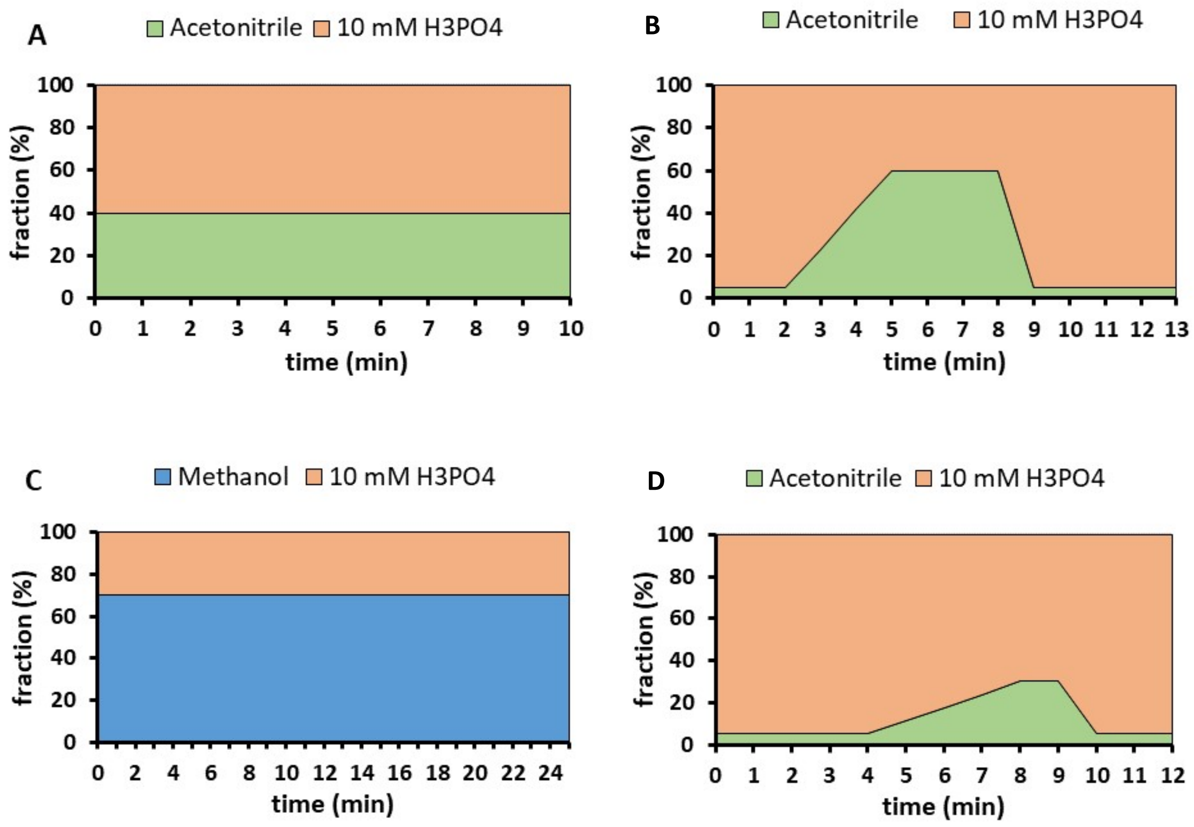

Figure S3: Eluent composition of the different HPLC/UV -methods for quantification of resorcinol, TMB, DPSe, DPDSe, *N*-acetylated-SeMet and *N*-acetylated-$SeCys_2$.

A: TMB quantification: Acetonitrile – 10 mM H_3PO_4 (isocratic); flow rate: 0.6 mL min^{-1}

B: Resorcinol and *N*-acetylated-SeMet quantification: Acetonitrile – 10 mM H_3PO_4 (gradient); flow rate: 0.8 mL min^{-1}

C: DPSe, DPDSe quantification: Methanol – 10 mM H_3PO_4 (isocratic); flow rate: 0.6 mL min^{-1}

D: *N*-acetylated-$SeCys_2$ quantification: Acetonitrile – 10 mM H_3PO_4 (soft gradient); flow rate: 0.8 mL min^{-1}

Text S6: Determination of limits of quantification for organic selenium compounds and associated reaction competitors

The limits of quantification (LOQ) for DMSe and DMDSe were calculated based on standard deviations of blanks relative to the calibration slope (Hubaux and Vos formula, equation S2), [24] while LOQs for DPSe, DPDSe, resorcinol and TMB were calculated based on the noise of the baseline relative to the signal of a standard (eq. S3). This method was not applied to *N*-acetylated-SeMet and *N*-acetylated-$SeCys_2$ due to an observed baseline drift. Instead, LOQs for these compounds were determined in an equivalent way compared to DMSe and DMDSe, but using a series of low-concentrated standard samples (eq. S4).

$LOQ_{DMSe,DMDSe} = 10 \times SD_{Blanks} / S_{cal}$ (equation S2)

where

- SD_{Blanks} is the standard deviation of the blanks (counts)
- S_{cal} is the slope of the calibration (counts per concentration unit)

$LOQ_{DPSe,DPDSe,Res,TMB} = 10 \times noise = 10 \times H_{noise}/H_{Standard} \times C_{Standard}$ (equation S3)

where

- H_{noise} is the height of the noise of the baseline (mAU)
- $H_{Standard}$ is the height of the noise of the lowest standard (mAU)
- $C_{Standard}$ is the concentration of the lowest standard (M)

$LOQ_{\textit{N}\text{-acetylated-SeMet}, \textit{N}\text{-acetylated-SeCys2}} = 10 \times SD_{standard}/S_{cal}$ (equation S4)

where

- $SD_{standard}$ is the standard deviation of the signal of a series of low-concentrated standards
- S_{cal} is the average signal for the target concentration

Text S7: Calculation of second-order rate constants for the reactions of organic selenium compounds with HOBr

Results from competition kinetics were analyzed by eqs. S5 and S6. [25]

Selenium species and resorcinol (for DPDSe: TMB) compete with each other for their reaction with HOBr (competition kinetics). Based on the fraction of resorcinol and the Se organic compound that reacted with HOBr, a slope is derived:

$$\ln(\frac{[Se]}{[Se]0}) = \ln(\frac{[Res]}{[Res]0})\frac{kapp,\ HOBr+Se}{kapp,HOBr+Res}$$ (equation S5)

$$slope = \frac{kapp,HOBr+Se}{kapp,HOBr+Res}$$

(equation S6)

where

- $[Se]_0$ is the initial concentration of the organic Se compound (before reaction)
- [Se] is the residual concentration of the organic Se compound (after reaction)
- $[Res]_0$ is the initial concentration of resorcinol (before reaction)
- [Res] is residual concentration of resorcinol (after reaction)
- k_{app}(HOBr+Se) is the apparent second-order rate constant for the reaction between HOBr and the organic Se compound

- k_{app}(HOBr+Res) is the apparent second-order rate constant for the reaction between HOBr and resorcinol

Multiplication of this slope with the apparent second-order rate constant for the reaction between resorcinol and HOBr results in the apparent second-order rate constant (k_{app}(Se+HOBr)) of the reaction between the organic Se compound and HOBr (eq. S7):

$$k_{app}(\text{HOBr+Se}) = \text{slope} \times k_{app}(\text{HOBr+Res}) \qquad \text{(equation S7)}$$

k_{app}(HOBr+Se) was calculated for pH 8 based on the slopes indicated in Tables S5 and S6, and equation S7.

Table S5: Slopes of competition kinetics experiments (reaction of the organic Se compound with HOBr in competition with the reaction of the competitor with HOBr). The slopes represent the ratios of the 2nd order rate constants (Se-HOBr vs competitor-HOBr reactivity).

Conditions: pH 8
Competitor: resorcinol or TMB
Buffer media:
$[PO_4]_{tot}$ = 20 mM for experiments with DMSe, DMDSe, DPSe and *N*-acetylated-SeMet
$[PO_4]_{tot}$ = 10 mM for experiments with DPDSe and *N*-acetylated-$SeCys_2$

	**DMSe**	**DMDSe**	**DPSe**	**DPDSe**	*N*-acetylated-**SeMet**	*N*-acetylated-**$SeCys_2$**
Replicate 1	4.1	2.6	2.0	3.0	13.8	2.1
Replicate 2	3.4	2.0	1.9	2.7	15.6	2.0
Replicate 3	4.0	2.4	1.8	2.8	16.7	2.2
Replicate 4	4.2	2.4				2.2
Replicate 5		2.4				
Replicate 6		2.1				
**average**	**3.9**	**2.3**	**1.9**	**2.8**	**15.3**	**2.1**
standard deviation	0.4	0.2	0.1	0.1	1.5	0.1

Table S6: Slopes of competition kinetics experiments performed in buffered artificial seawater and perchlorate medium (= high ionic strength) medium. The slopes represent the ratios of the 2nd order rate constants (Se-HOBr vs competitor-HOBr reactivity).
Conditions: pH 8
Competitor: resorcinol
Buffer media:
$DMSe_{seawater}$: $[PO_4]_{tot}$ = 20 mM, [NaCl] = 0.55 M, [KBr] = 840 µM
$DMDSe_{seawater}$: $[PO_4]_{tot}$ = 20 mM, [NaCl] = 0.55 M, [KBr] = 840 µM
$DMSe_{seawater\ (without\ Br-)}$: $[PO_4]_{tot}$ = 20 mM, [NaCl] = 0.55 M
$DMSe_{perchlorate}$: $[PO_4]_{tot}$ = 20 mM, $[NaClO_4]$ = 0.55 M

The slope value for DMSe,seaw (26.0±2.8) is far beyond 10 and therefore not ideal for an exact quantification of kDMSe+HOBr in seawater medium. We used resorcinol and not sulfite (with a higher rate constant) because of sulfite oxidation by the DMSe oxidation product (i.e. DMSeO). Still, the decrease of resorcinol was large enough to enable a precise quantification by HPLC/UV. The coefficient of determination (R^2) for the two replicates was 0.95 and 0.96

	**$DMSe_{seaw}$**	**$DMDSe_{seaw}$**	**$DMSe_{seaw\ (without\ Br-)}$**	**$DMSe_{perchl.}$**
Replicate 1	24.0	2.5	7.3	13.7
Replicate 2	28.0	2.4		7.1
**average**	**26.0**	**2.5**	**7.3**	**10.5**
standard deviation	2.8	0.1	-	3.3

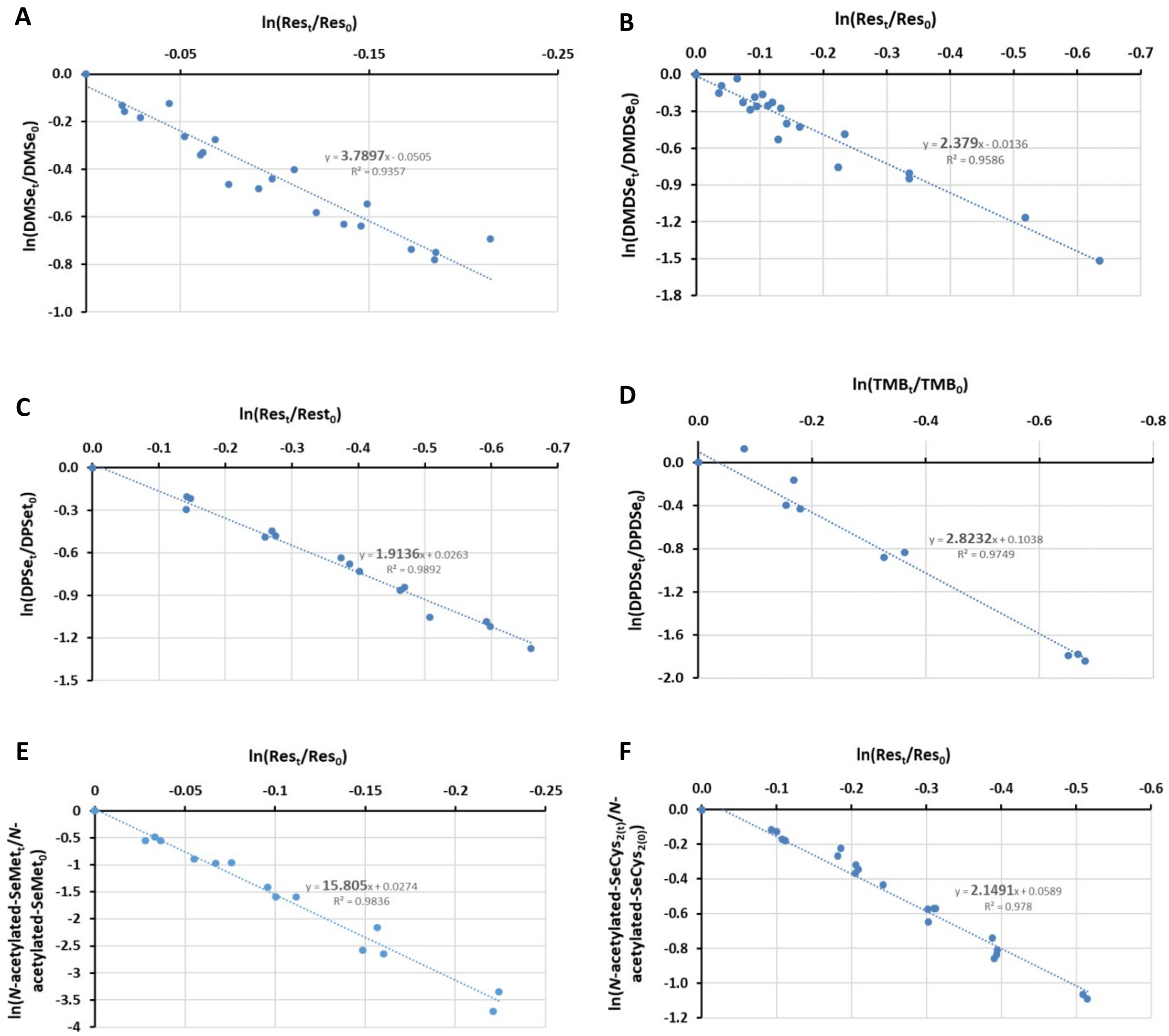

Figure S4: Competition kinetics plots of the ln of the relative residual concentrations of target organic Se compounds and competitors from kinetic experiments with HOBr performed in phosphate-buffered medium at pH 8. The plots include all data points from all replicates (Table S5) and the slopes represent the average slopes from all experiments. Average slope values are slightly different compared to Table S5 because in this figure the data points are fitted to a linear regression, representing the slopes in equations S5 - S7 and not calculated as an average as in Table S5. (A) DMSe, (B) DMDSe, (C) DPSe, (D) DPDSe, (E) *N*-acetylated-SeMet, (F) *N*-acetylated-SeCys$_2$. Conditions: pH 8, competitor: resorcinol (Res) or TMB as indicated at the X-axes.

Buffer media:

$[PO_4]_{tot}$ = 20 mM for experiments with DMSe, DMDSe, DPSe and *N*-acetylated-SeMet

$[PO_4]_{tot}$ = 10 mM for experiments with DPDSe and *N*-acetylated-SeCys$_2$

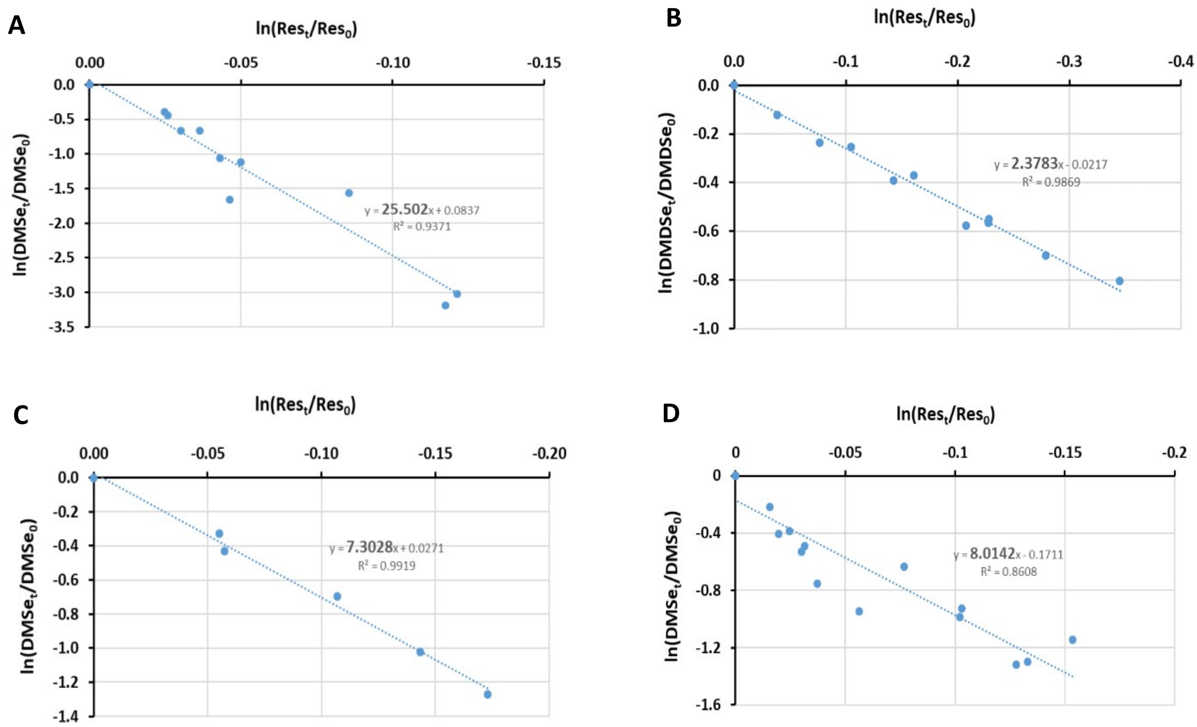

Figure S5: Competition kinetics plots of ln of the measured relative residual concentrations of DMSe, DMDSe and competitors from kinetic experiments with HOBr performed in buffered artificial seawater medium/high ionic strength medium at pH 8. The plots include all data points and derive an average slope of all data from different experiments. The average slope values are slightly different compared to Table S6 because in this figure data points are fitted to a linear regression, representing the slopes in equations S5 - S7 and not calculated as an average as in Table S6. (A) DMSe in buffered seawater medium, (B) DMDSe in buffered seawater medium, (C) DMSe in buffered seawater medium without Br^-, (D) DMSe in buffered perchlorate medium with the same ionic strength as the seawater medium.

Conditions: pH 8

Competitor: resorcinol

Buffer media:

$DMSe_{seawater}$: $[PO_4]_{tot}$ = 20 mM, [NaCl] = 0.55 M, [KBr] = 840 µM

$DMDSe_{seawater}$: $[PO_4]_{tot}$ = 20 mM, [NaCl] = 0.55 M, [KBr] = 840 µM

$DMSe_{seawater\ (without\ Br-)}$: $[PO_4]_{tot}$ = 20 mM, [NaCl] = 0.55 M

$DMSe_{perchlorate}$: $[PO_4]_{tot}$ = 20 mM, [Na-perchlorate] = 0.55 M

Text S8: Identification and (semi)quantification of Se-containing oxidation products by HR-MS

Preliminary HR-MS analyses demonstrated that the Se-containing oxidation products were either not ionized in negative ion mode or were ionized in both negative and positive ion modes (e.g., *N*-acetylated-SeMet). All analyses were thus performed in positive ion mode (direct infusion) with the following settings: syringe pump flow rate: 10 µL min^{-1}, ESI spray voltage: +3.0 kV; sheath gas flow rate: 5; capillary temperature: 320 °C; S-lens RF level: 55; automatic gain control: 1×10^6 (maximum

accumulation time of 50 ms), and resolution: 240’000. Samples were loaded to the ESI source using the instrument’s built-in syringe pump and a Hamilton syringe (500 µL) connected to the ESI source via PEEK tubing.

To identify the Se-containing oxidation products by looking for the Se isotopic pattern, full mass spectra with different *m/z* ranges (e.g., 60-1000, 60-200, 200-400, 400-600) were recorded for all samples. For the compound identification with the software Freestyle (Thermo Scientific), the “predict composition” function was used with a mass tolerance of 5 ppm and allowing for the presence of N, O, C, H, S, Cl, P, Na, K, Br and Se. For the semi-quantification of the identified Se-containing oxidation products, single ion mode (SIM) spectra at the nominal mass ± 0.2 were recorded.

The notation of the detected masses of the identified organic Se compounds/oxidation products as well as the calculation of the mass accuracy (Δppm**;** equation S8) follow the recommendation by Brenton and Godfrey (2010). [26]

$$\Delta ppm = \frac{(m_i - m_a)}{m_a} . 10^6 \qquad \text{(equation S8)}$$

where

- Δppm represents the mass accuracy (in parts per million) and can be positive or negative;
- m_i is the measured *m/z;* and m_a is the calculated (theoretical) *m/z* obtained using the Eawag Web-interface “enviPath”.[27]

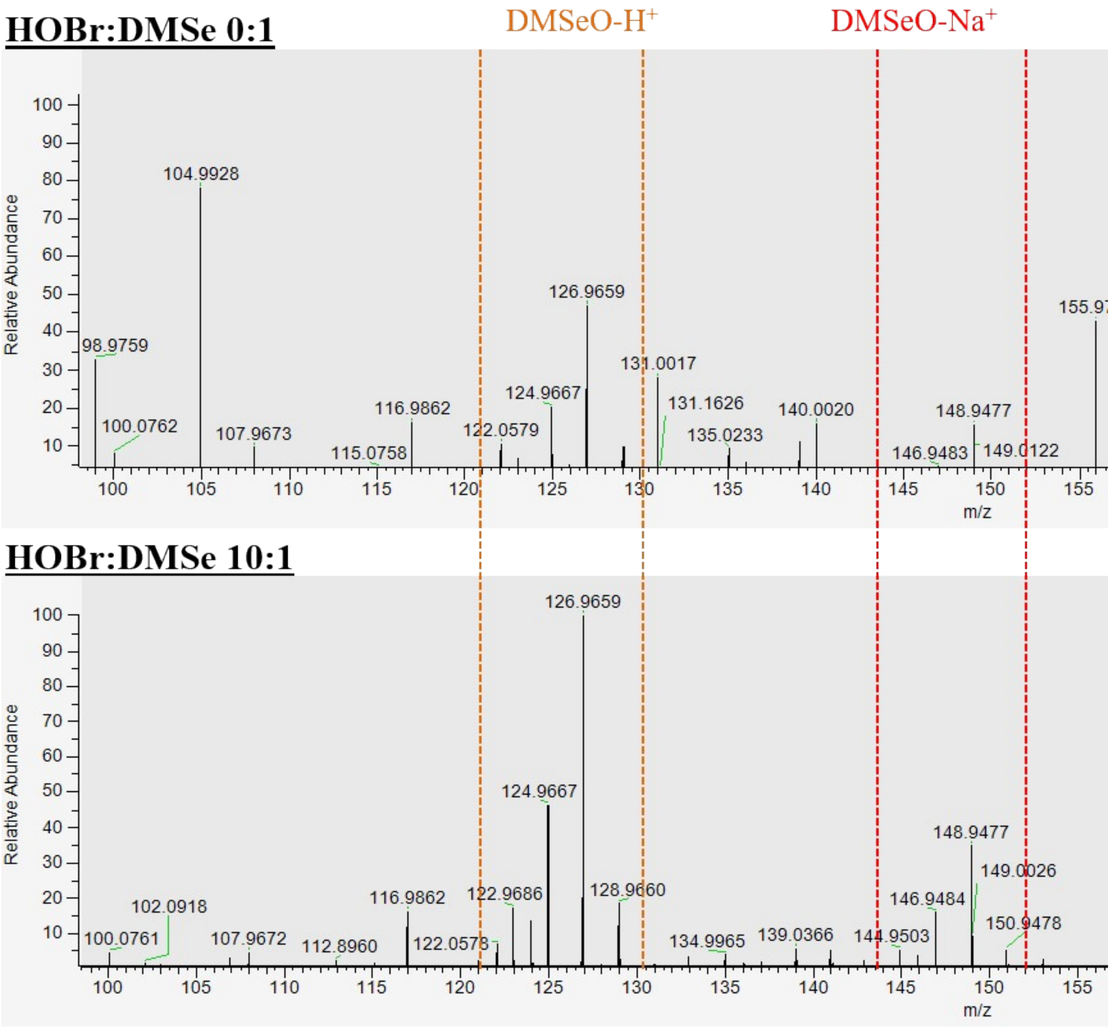

Figure S6: HR-MS mass spectra (between *m/z* 100 and 155) for solutions after the reaction between DMSe and HOBr allowing for the identification of DMSeO as the Se-containing oxidation product (detected with Na^+ -*m/z* 148.9476- and H^+ -*m/z* 126.9658- adducts and mass accuracy between -0.7 and 1 ppm). The space between the stippled lines indicates the *m/z* area of the Se isotopic pattern (excluding ^{74}Se which is of low abundance) for the identified compounds. Experimental conditions: pH 8, [$NaHCO_3$] = 1 mM, [DMSe] = 6.25 µM, [HOBr] = 0 – 62.5 µM.

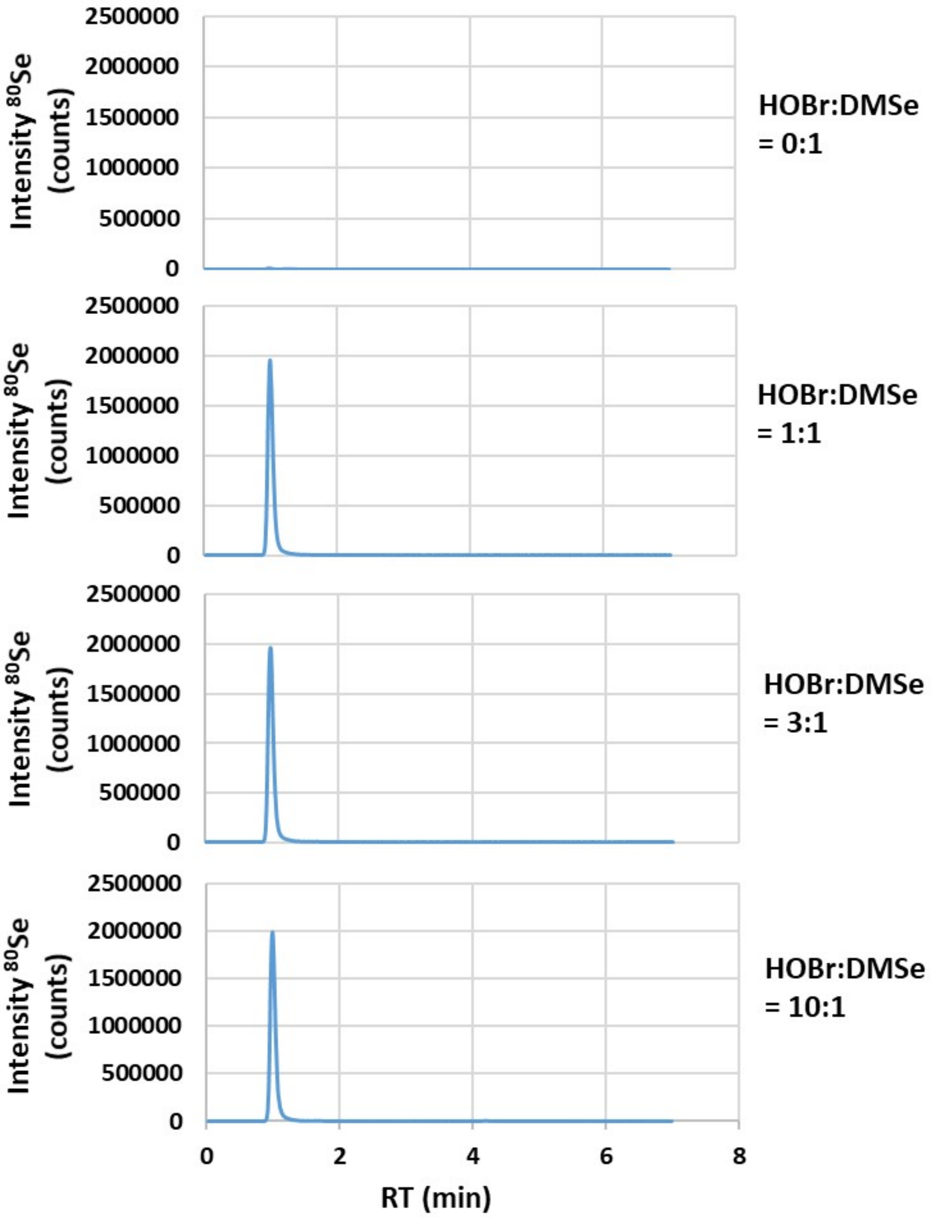

Figure S7: LC-ICP-MS/MS chromatograms indicating ^{80}Se counts for solutions obtained after the reaction between DMSe and HOBr. Only one Se peak is detected at molar HOBr:DMSe ratios between 1:1 and 10:1. Neither Se(IV) nor Se(VI) were detected.

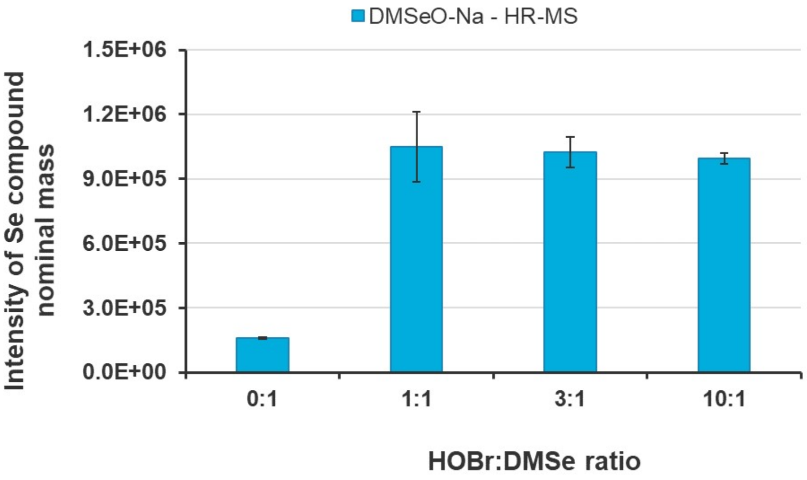

Figure S8: Semi-quantitative HR-MS data for the product of the reaction between DMSe and HOBr, i.e., DMSeO. The intensity of DMSeO remains constant between molar HOBr:DMSe ratios of 1:1 to 10:1, indicating that DMSeO is not further oxidized by HOBr. Experimental conditions: pH 8, [$NaHCO_3$] = 1 mM, [DMSe] = 6.25 µM (= 500 µg/L Se), [HOBr] = 0 – 62.5 µM. The 0:1 ratio represents a blank experiment of DMSe, i.e. no addition of HOBr.

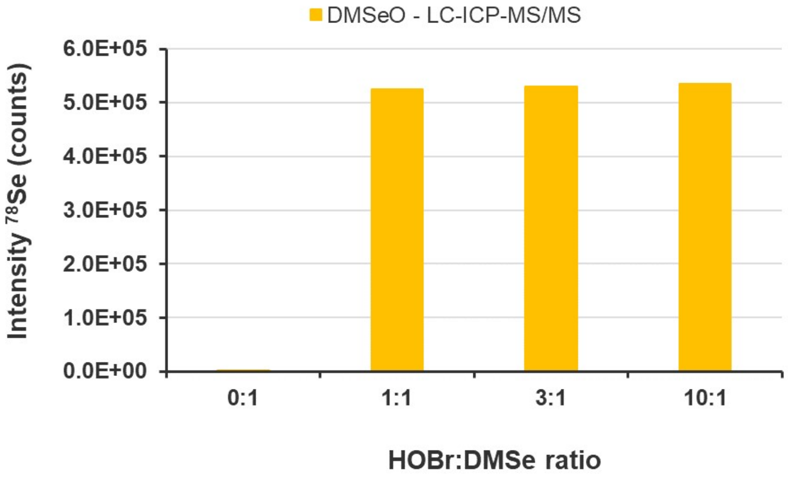

Figure S9: Semi-quantitative LC-ICP-MS/MS data for DMSeO, confirming the HR-MS data in Figure S8. It should be noted that the intensities obtained by HR-MS (Figure S8) and LC-ICP-MS/MS are not directly comparable (as these are two different instruments). Experimental conditions: pH 8, [$NaHCO_3$] = 1 mM, [DMSe] = 6.25 µM (= 500 µg/L Se), [HOBr] = 0 – 62.5 µM.

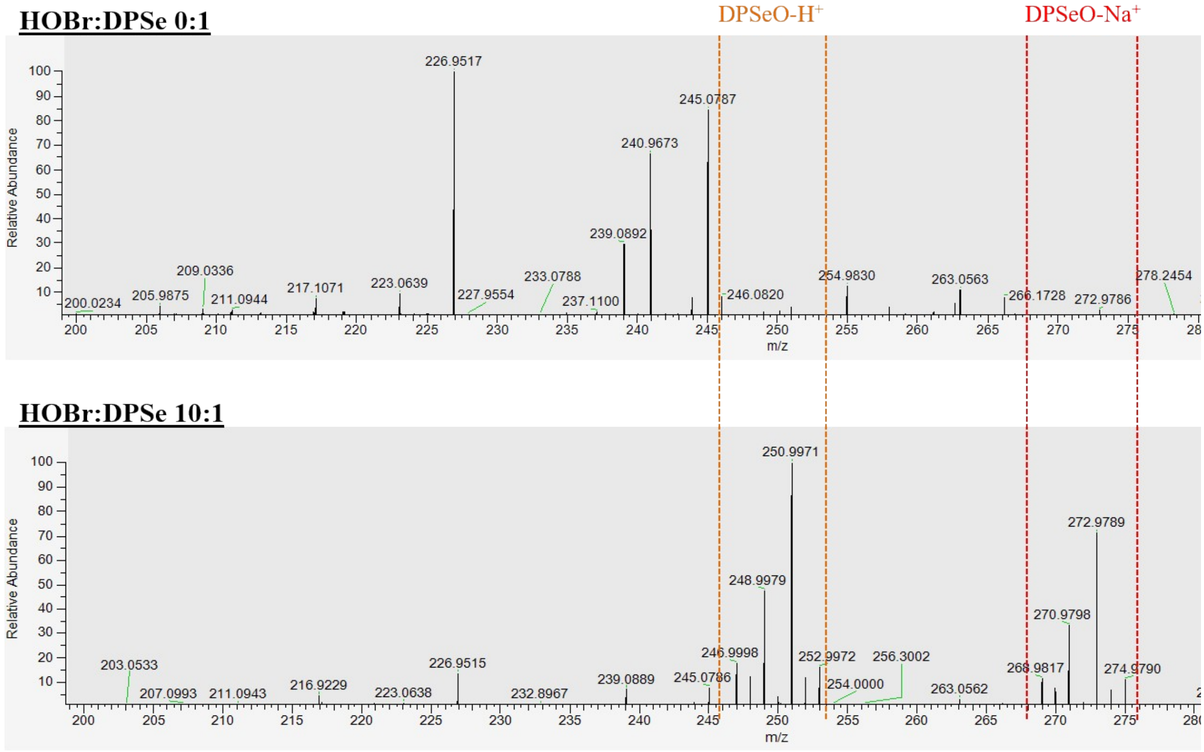

Figure S10: HR-MS mass spectra (between *m/z* 200 and 280) for solutions after the reaction between DPSe and HOBr (two molar ratios) allowing for the identification of DPSeO as the Se-containing oxidation product (detected with Na^+ -*m/z* 272.9788- and H^+ -*m/z* 250.9970- adducts and mass accuracy between -0.4 and 0.1 ppm). The space between the stippled lines indicates the *m/z* area of the Se isotopic pattern (excluding ^{74}Se, which is of low abundance) for the identified compounds. Experimental conditions: pH 8, [$NaHCO_3$] = 1 mM, [DPSe] = 6.25 µM, [HOBr] = 0 – 62.5 µM.

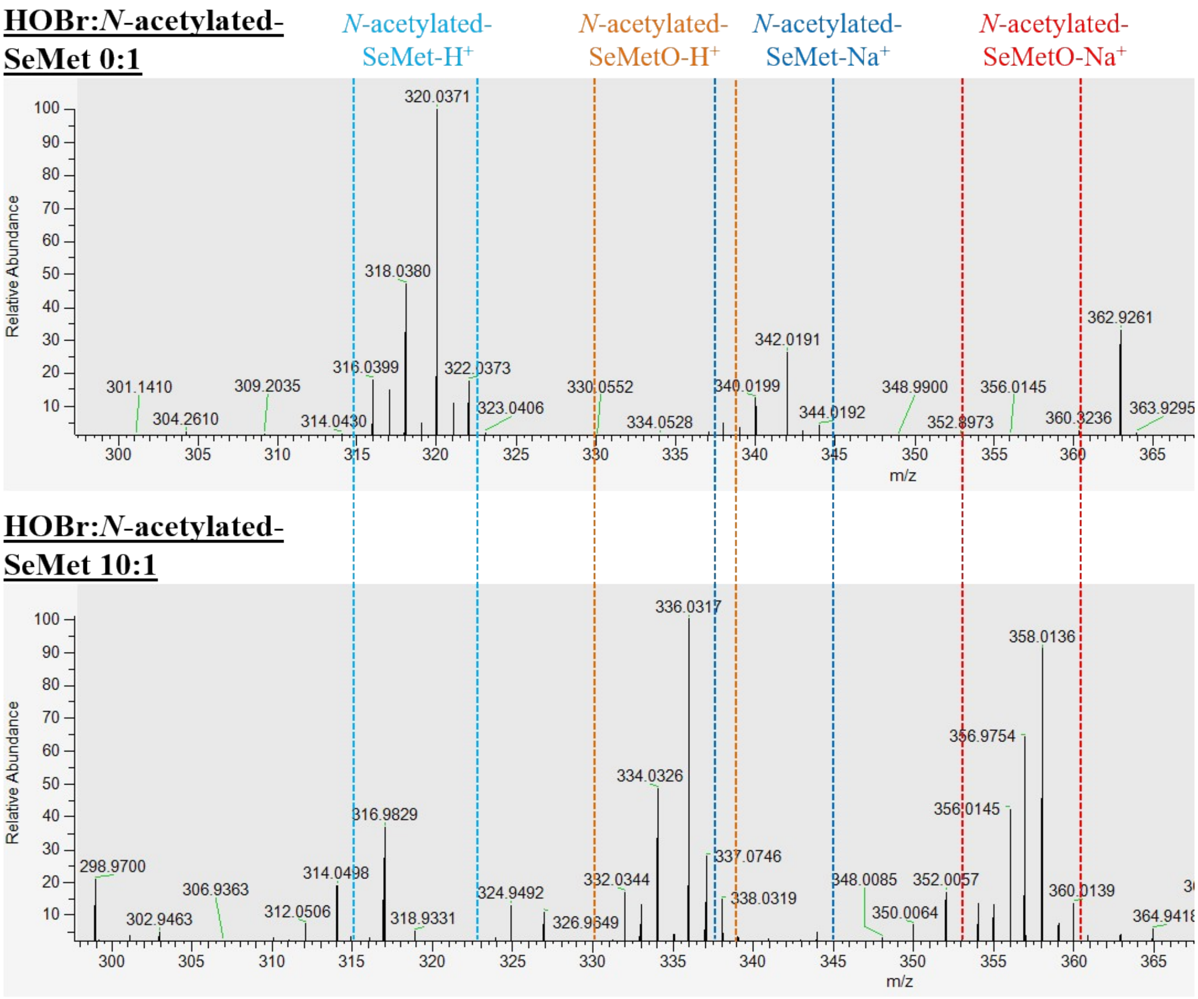

Figure S11: HR-MS mass spectra (between *m/z* 300 and 365) for solutions after the reaction of *N*-acetylated-SeMet and HOBr (two molar ratios) allowing for the identification of *N*-acetylated-SeMetO as the Se-containing oxidation product (detected with Na^+ -*m/z* 336.0318 - and $2Na^+$-H^+ -*m/z* 358.0137- adducts and mass accuracy between -0.9 and -0.8 ppm). *N*-acetylated-SeMet was detected with Na^+ -*m/z* 320.0372 - and $2Na^+$-H^+ -*m/z* 342.0191- adducts and mass accuracy between -0.5 and -0.3 ppm). The space between the stippled lines indicates the *m/z* area of the Se isotopic pattern (excluding ^{74}Se, which is of low abundance) for the identified compounds. Experimental conditions: pH 8, [$NaHCO_3$] = 1 mM, [*N*-acetylated-SeMet] = 6.25 µM, [HOBr] = 0 – 62.5 µM.

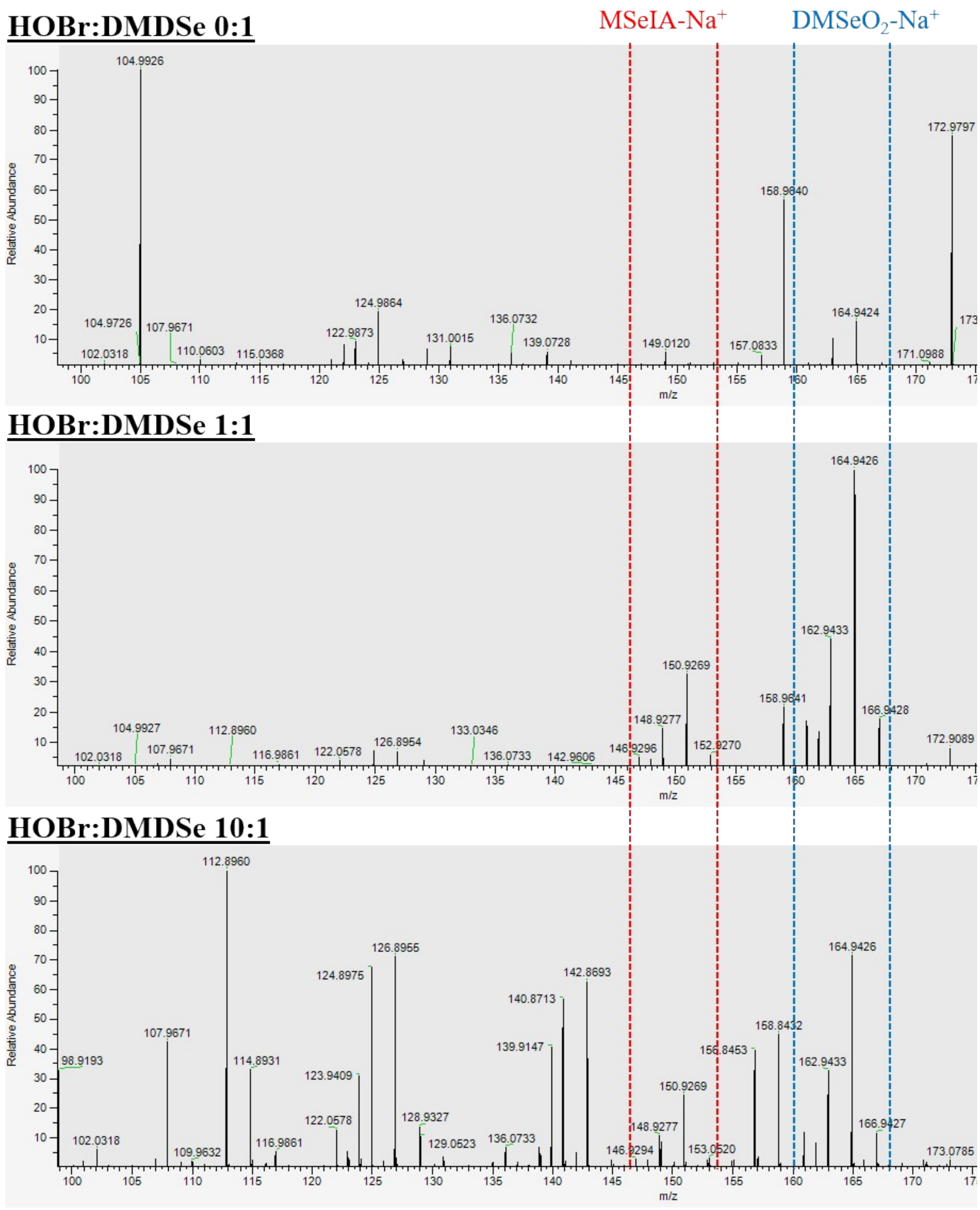

Figure S12: HR-MS mass spectra (between *m/z* 100 and 170) for solutions after the reaction between DMDSe and HOBr (two molar ratios) allowing for the identification of methane seleninic acid (MSeIA) as one Se-containing oxidation product (detected with Na^+ adduct -*m/z* 150.9268- and mass accuracy between -1 and 0.5 ppm). The space between the stippled lines indicates the *m/z* area of the Se isotopic pattern (excluding ^{74}Se, which is of low abundance) for the identified compounds. Experimental conditions: pH 8, [$NaHCO_3$] = 1 mM, [DMDSe] = 6.25 µM, [HOBr] = 0 – 62.5 µM. Besides MSeIA, $DMSeO_2$ was also detected by HR-MS (Na^+ adduct - *m/z* 164.9425- and mass accuracy between -0.9 and -0.4 ppm).

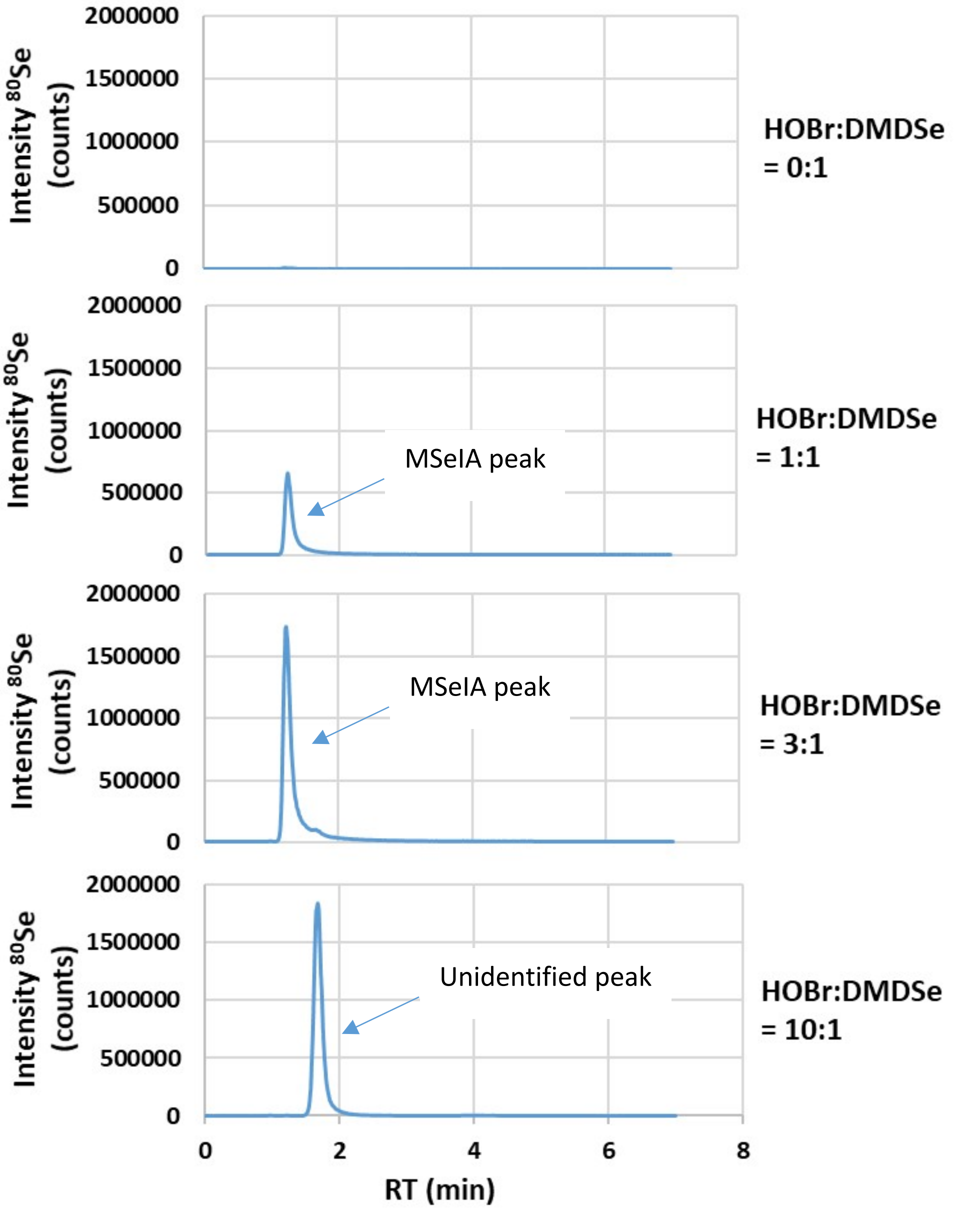

Figure S13: LC-ICP-MS/MS chromatograms obtained for solutions after the reaction between DMDSe and HOBr (four molar ratios), which (i) confirm the formation of MSeIA (identified using a MSeIA standard) observed with HR-MS at molar HOBr:DMDSe ratios of 1:1 and 3:1; and (ii) show the reaction of MSeIA with HOBr and the formation of another Se compound that could not be identified with HR-MS. Neither Se(IV) nor Se(VI) were detected under any tested reaction conditions.

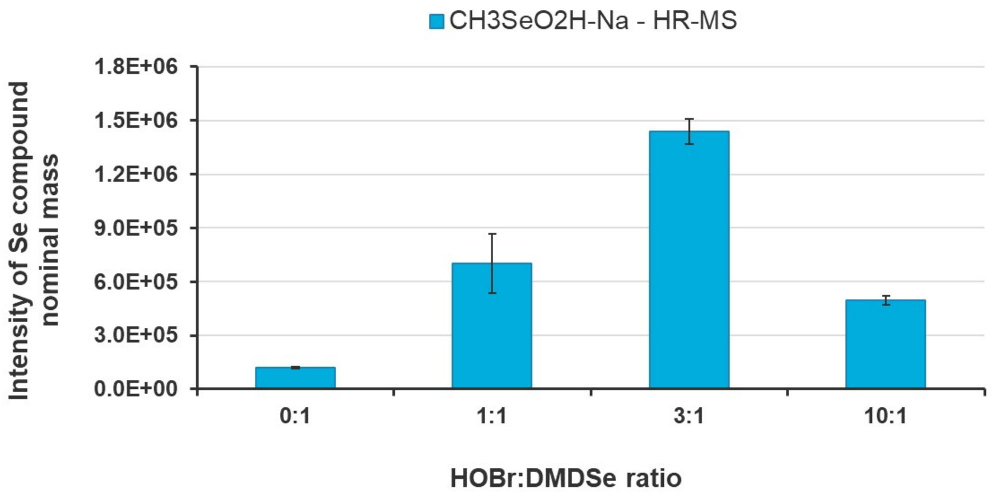

Figure S14: Semi-quantitative HR-MS data for the reaction between DMDSe and HOBr (four molar ratios; 0:1 blank), showing that the highest yield of MSeIA (CH_3SeO_2H) occurred at the HOBr:DMDSe = 3:1 ratio. Experimental conditions: pH 8, [$NaHCO_3$] = 1 mM, [DMDSe] = 6.25 µM (= 1000 µg/L Se), [HOBr] = 0 – 62.5 µM.

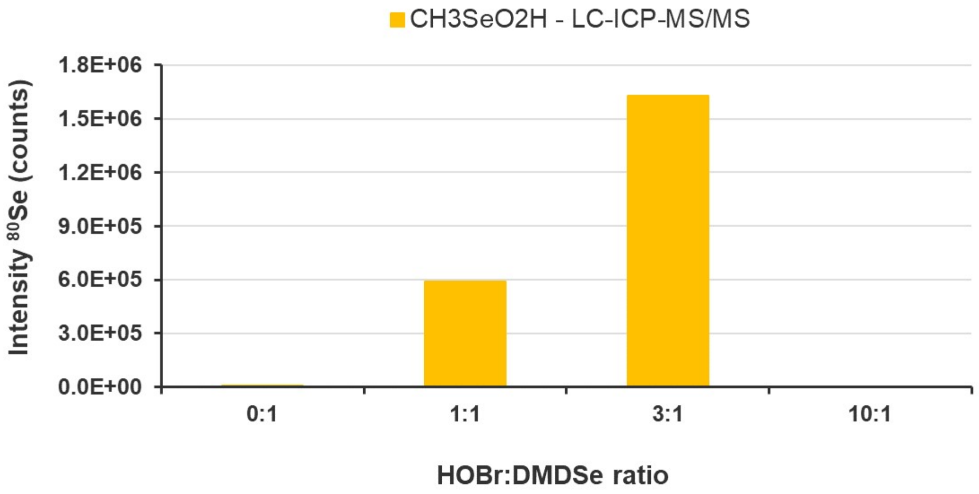

Figure S15: Semi-quantitative LC-ICP-MS/MS data for the reaction between DMDSe and HOBr (four molar ratios; 0:1 blank), showing that the highest yield of MSeIA (CH_3SeO_2H) occurred at the HOBr:DMDSe = 3:1 ratio. Note that the intensities obtained by HR-MS and LC-ICP-MS/MS are not comparable (as these are two different instruments). Experimental conditions: pH 8, [$NaHCO_3$] = 1 mM, [DMDSe] = 6.25 µM (= 1000 µg/L Se), [HOBr] = 0 – 62.5 µM.

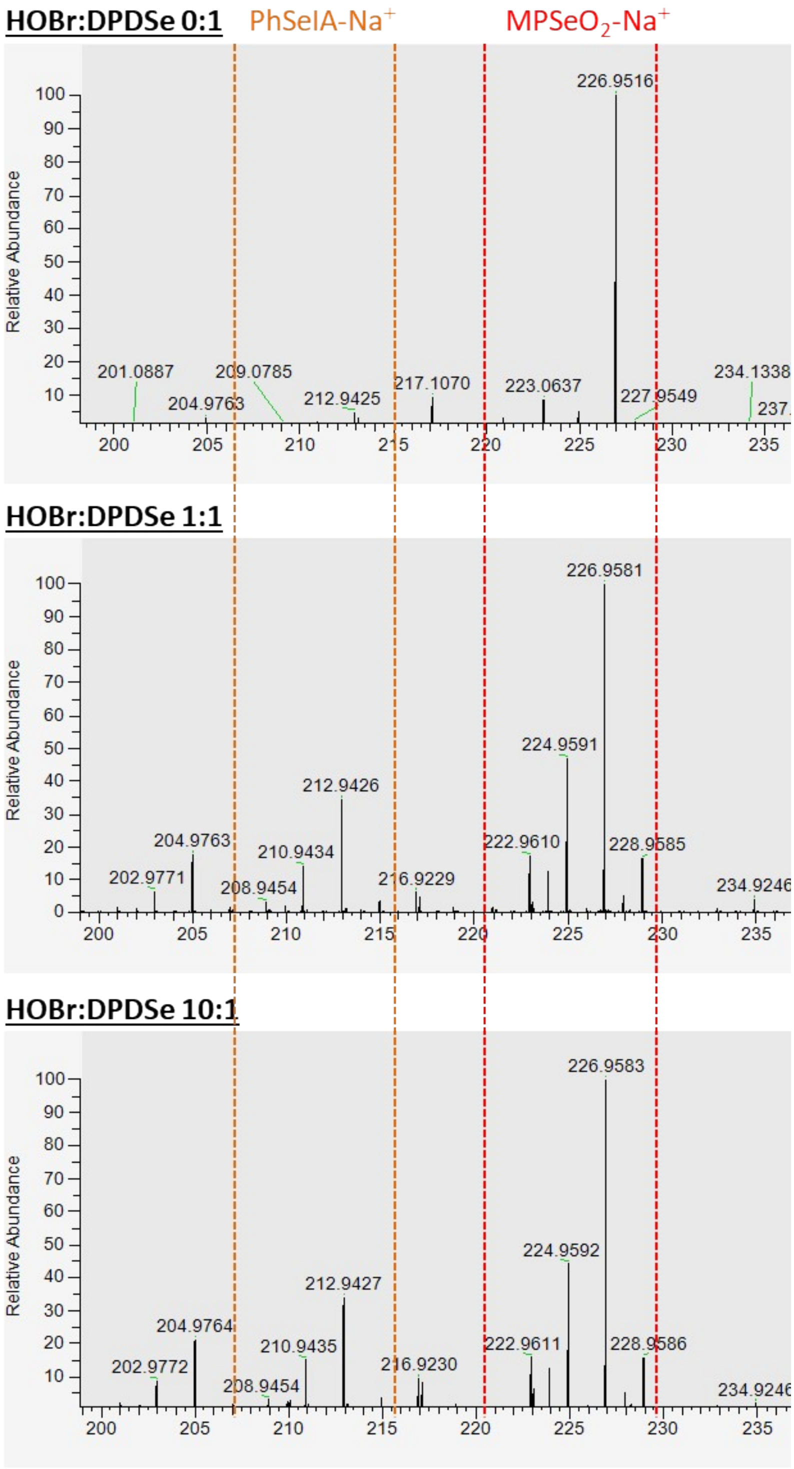

Figure S16: HR-MS mass spectra for solutions after the reaction between DPDSe and HOBr (two molar ratios) allowing for the identification of phenyl seleninic acid (PhSeIA, $C_6H_5SeO_2H$, detected with Na^+ - *m/z* 212.9424- adducts and mass accuracy between -0.6 and 0.8 ppm) and methyl phenyl selenone ($MPSeO_2$, $C_7H_7SeO_2$, detected with Na^+ - *m/z* 226.9582- adducts and mass accuracy between -0.3 and 0.6 ppm) as two Se-containing oxidation products. Experimental conditions: pH 8, [$NaHCO_3$] = 1 mM, [DPDSe] = 6.25 µM, [HOBr] = 0 – 62.5 µM.

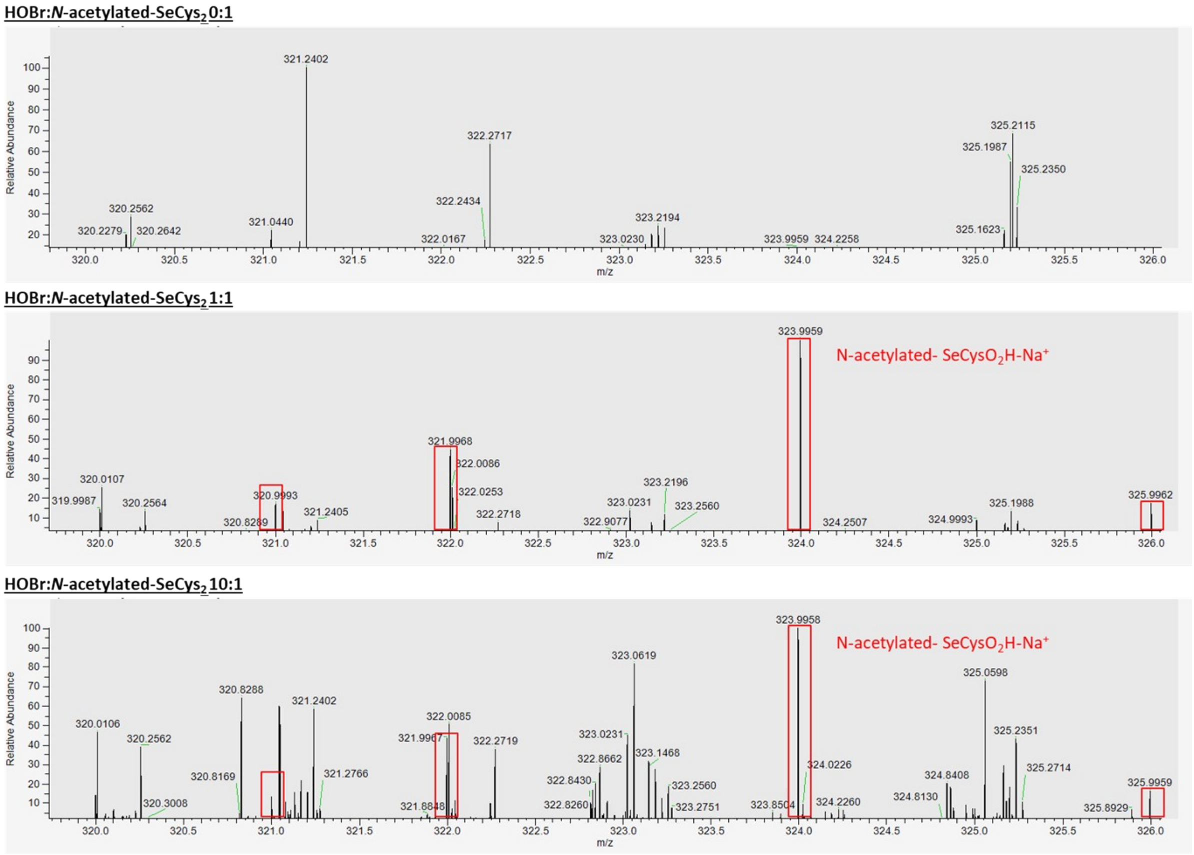

Figure S17: HR-MS mass spectra for solutions after the reaction between *N*-acetylated-SeCys$_2$ and HOBr (three molar ratios) allowing for the identification of *N*-acetylated-SeCysO$_2$H (detected with Na$^+$ - *m/z* 323.9956- and 2Na$^+$-H$^+$ -*m/z* 345.9776- adducts and mass accuracy between -0.2 and 1 ppm) as a Se-containing oxidation product (only the MS spectra of the Na$^+$ adduct of *N*-acetylated-SeCysO$_2$H -*m/z* 323.9956- is shown). *N*-acetylated-SeCys$_2$ was detected with Na$^+$ -*m/z* 559.0068 - and 2Na$^+$-H$^+$ -*m/z* 580.9888- adducts and mass accuracy between 0.6 and 1.4 ppm).

Experimental conditions: pH 8, [NaHCO$_3$] = 1 mM, [*N*-acetylated-SeCys$_2$] = 6.25 µM, [HOBr] = 0 – 62.5 µM

476

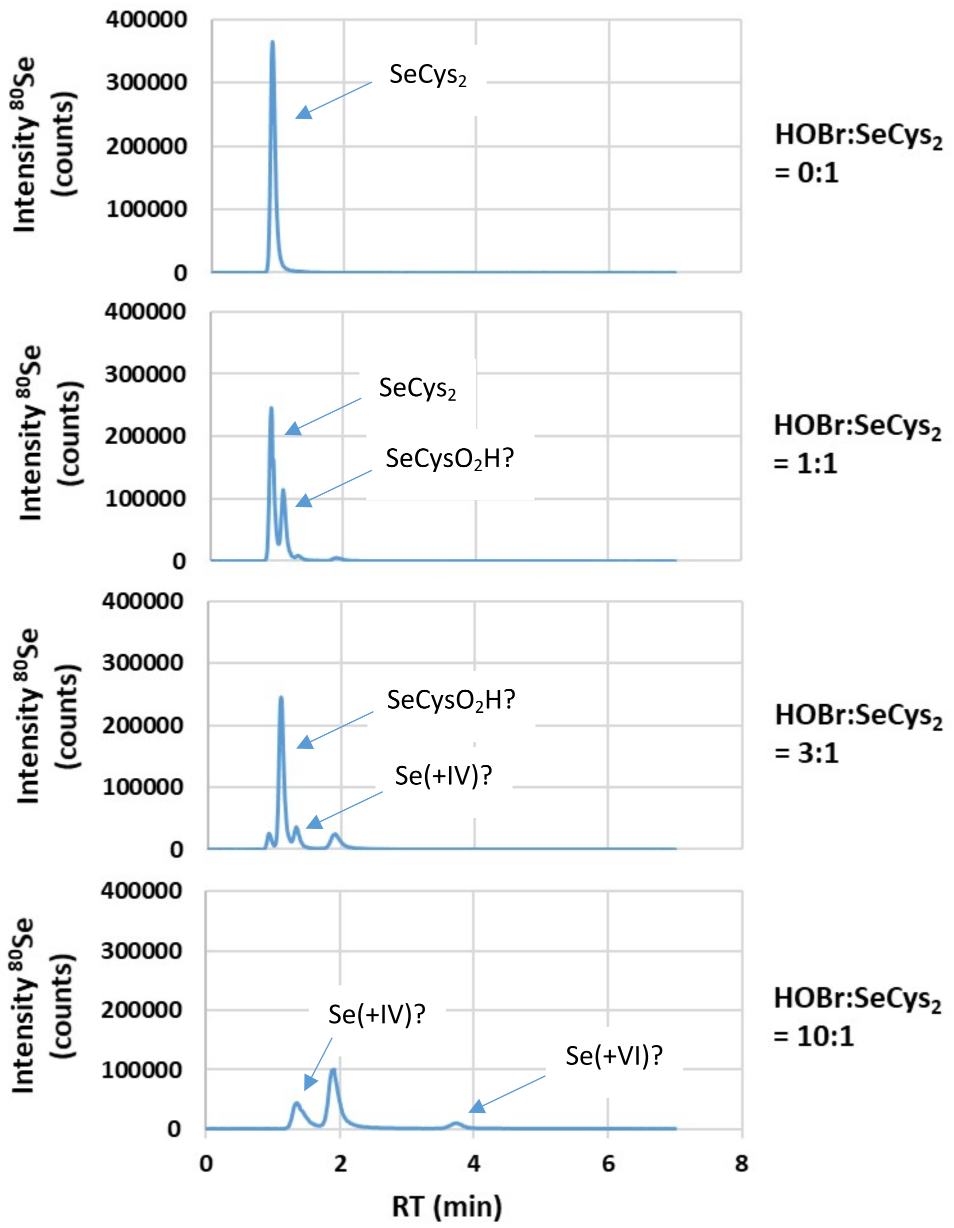

Figure S18: LC-ICP-MS/MS chromatograms indicating ^{80}Se counts for solutions obtained after the reaction between *N*-acetylated-$SeCys_2$ (in the figure displayed as $SeCys_2$) and HOBr for four molar ratios. $SeCys_2$ elutes at 0.913 min. The first oxidation product appears at a RT of 1.1 min (HOBr:$SeCys_2$ ratio of 1:1), which becomes predominant at a molar HOBr:$SeCys_2$ ratio of 3:1. At a molar HOBr:$SeCys_2$ ratio of 3:1, there is only a small residual concentration of $SeCys_2$ and 2 other minor oxidation products are visible, i.e., at RT = 1.36 min, which is potentially Se(+IV), and at RT = 1.9 min. At the HOBr:$SeCys_2$ = 10:1 ratio, the main oxidation product appears at RT = 1.9 min followed by the one at RT = 1.36 min. Furthermore, a third product appears at RT = 3.8 min (potentially Se[+VI]).

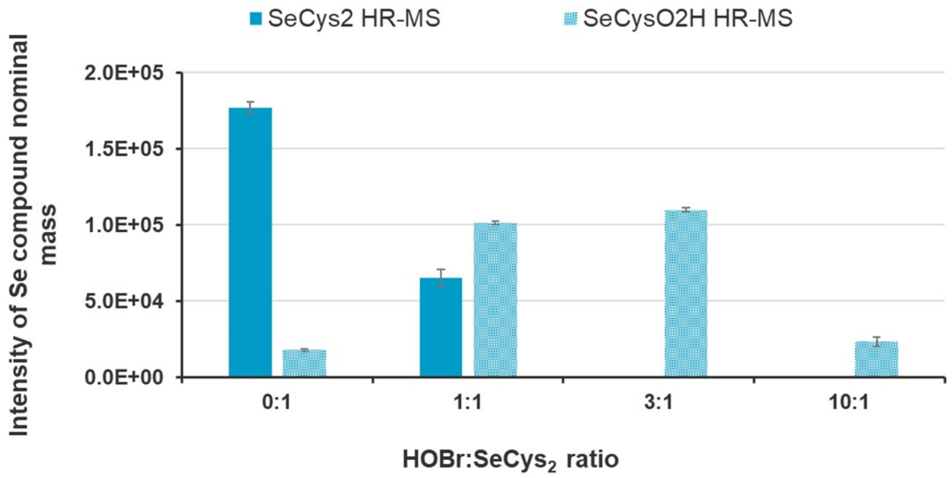

Figure S19: Semi-quantitative HR-MS data for the product of the reaction between *N*-acetylated-SeCys$_2$ (in the figure displayed as SeCys$_2$) and HOBr (four molar ratios; 0:1 blank), showing that the intensity of SeCys$_2$ decreased from the molar HOBr:SeCys$_2$ ratio of 0:1 to 3:1 to zero. HR-MS data show that SeCys$_2$ is transformed to Selenocysteine-seleninic-acid (SeCysO$_2$H) at molar HOBr:SeCys$_2$ ratios of 1:1 and 3:1, which is further oxidized at a molar HOBr:SeCys$_2$ ratio of 10:1. Experimental conditions: pH 8, [NaHCO$_3$] = 1 mM, [*N*-acetylated-SeCys$_2$] = 6.25 µM (= 1000 µg/L Se), [HOBr] = 0 – 62.5 µM.

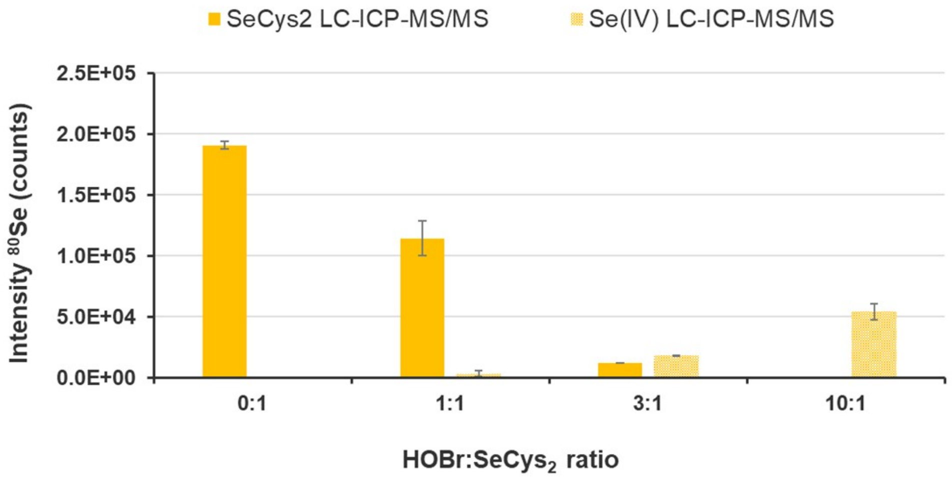

Figure S20: Semi-quantitative LC-ICP-MS/MS data for the reaction between *N*-acetylated-SeCys$_2$ (in the figure displayed as SeCys$_2$) and HOBr (four molar ratios; 0:1 blank), showing that the intensity of SeCys$_2$ decreased from the molar HOBr:SeCys$_2$ ratio of 0:1 to 3:1 to almost zero. LC-ICP-MS/MS data suggests that SeCysO$_2$H is transformed to Se(+IV) or Se(+VI), since the intensity for Se(+IV) is highest for the molar HOBr:SeCys$_2$ ratio of 10:1 and a new peak appeared in the associated LC-ICP-MS/MS chromatogram provided in Figure S18 (at RT = 3.8 min), which is potentially Se(+VI). Experimental conditions: pH 8, [NaHCO$_3$] = 1 mM, [*N*-acetylated-SeCys$_2$] = 6.25 µM (= 1000 µg/L Se), [HOBr] = 0 – 62.5 µM.

Text S9: Experiments investigating the higher reactivity of DMSe with HOBr in buffered artificial seawater medium compared to buffered ultrapure water

A separate kinetic experiment for the DMSe-HOBr reaction in buffered artificial seawater in absence of Br^- (i.e. $[PO_4]_{tot}$ = 20 mM, [NaCl] = 0.55 M) resulted in a substantially lower DMSe-HOBr reactivity ($k_{DMSe+HOBr} = 1.3 \times 10^8$ M^{-1} s^{-1}) in comparison to the buffered artificial seawater in presence of Br^- (k = $4.7 \pm 0.5 \times 10^8$) but still higher than in the sample with buffered ultrapure water. In another experiment, the same ionic strength was applied with perchlorate ($[PO_4]_{tot}$ = 20 mM, $[NaClO_4]$ = 0.55 M), which yielded a similar reactivity compared to the Br^- free seawater medium ($k_{DMSe+HOBr} = (2.2 \pm 0.7) \times 10^8$ M^{-1} s^{-1}) (Table S6, Figure S5). The higher slope values (Figure S5) obtained for higher ionic strength are not caused by slower HOBr-resorcinol kinetics under a higher ionic strength. A possible change of pK_a towards lower values would shift resorcinol speciation towards the more reactive deprotonated species and HOBr to the less reactive OBr^- (Table S4). Based on this, it can be concluded that a higher ionic strength contributes to a higher DMSe-HOBr reactivity. However, it is also evident that Br^- contributes to a higher reactivity of the DMSe-HOBr reaction, since the reactivity in a Br^--containing artificial seawater medium ($k_{DMSe+HOBr} = (5.4 \pm 0.6) \times 10^8$ M^{-1} s^{-1}) is significantly different to the reactivity in a Br^--free seawater medium ($k_{DMSe+HOBr} = 1.5 \times 10^8$ M^{-1} s^{-1}) or in a perchlorate medium ($k_{DMSe+HOBr}$ = $(2.2 \pm 0.7) \times 10^8$ M^{-1} s^{-1}). We do not have a reasonable explanation for how Br^- enhances the reactivity. An indirect effect of Br^- via the formation of other reactive bromine species (e.g. Br_2, via the reaction between Br^- and HOBr) is not expected to be the reason for the higher observed reactivity of DMSe in buffered artificial seawater medium. Concentrations of reactive bromine species can be calculated based on concentrations of halides (i.e. Br^-, Cl^-), HOBr and H^+ used in kinetic experiments (Table S7), and equations S9 - S11.

$HOBr + Br^- + H^+ \rightleftharpoons Br_2 + H_2O$ K = 1.64×10^8 M^{-2} [29] (S9)

$HOBr + Cl^- + H^+ \rightleftharpoons BrCl + H_2O$ K = 7692 M^{-2} [30] (S10)

$2\ HOBr \rightleftharpoons Br_2O + H_2O$ K = 6.31 M^{-1} [31] (S11)

Normalization of calculated concentrations of Br_2, BrCl and Br_2O to HOBr concentrations results in their respective mole fractions (Table S8).

Calculated Br_2 concentrations (based on equation S9) account for only 0.14% of HOBr (Table S8), which could increase the observed reactivity in artificial seawater medium compared to the phosphate-buffered medium by 40% at most, considering an upper reactivity limit of ≈2×10^{10} M^{-1} s^{-1} for second-order reactions (diffusion limit). This cannot explain the 6.5-fold higher reactivity of DMSe in buffered artificial seawater medium than phosphate-buffered medium.

Table S7: Concentrations of halides, HOBr and H^+ in DMSe-HOBr experiments performed in buffered artificial seawater.

**Compound**	**Concentration in experiment [M]**	**Remark**
Chloride (Cl^-)	0.55	
Bromide (Br^-)	≥8.4×10^{-4}	Bromide from KBr and HOBr stock solution. $[Br^-_{tot}] = 8.4\times10^{-6}$ M + 1.34 × [HOBr]
HOBr	0 - 1.5×10^{-6}	
H^+	1×10^{-8}	

Table S8: Concentrations and mole fractions of BrCl, Br_2O and Br_2 for used HOBr concentrations in buffered artificial seawater medium according to Table S7 at pH 8.

	**BrCl**		**Br_2O**		**Br_2**	
	[M]	% of total bromine	[M]	% of total bromine	[M]	% of total bromine
HOBr 0 µM	0		0		0	
HOBr 5 µM	2.1×10^{-10}	**4.23×10^{-3}**	1.58×10^{-10}	**3.16×10^{-3}**	6.94×10^{-9}	**1.39×10^{-1}**
HOBr 10 µM	4.2×10^{-10}	**4.23×10^{-3}**	6.31×10^{-10}	**6.31×10^{-3}**	1.40×10^{-8}	**1.40×10^{-1}**
HOBr 15 µM	6.3×10^{-10}	**4.23×10^{-3}**	1.42×10^{-9}	**9.47×10^{-3}**	2.12×10^{-8}	**1.41×10^{-1}**
HOBr 125 µM	5.3×10^{-9}	**4.23×10^{-3}**	9.86×10^{-8}	**7.89×10^{-2}**	2.07×10^{-7}	**1.65×10^{-1}**

Text S10: Kinetics of the HOBr-BOC_2O reaction

A decrease of HOBr concentration was observed when it was exposed to BOC_2O. To assess the importance of the HOBr-BOC_2O reaction, kinetic experiments under pseudo first-order conditions in excess of BOC_2O were performed. BOC_2O was mixed with HOBr in a molar ratio of BOC_2O:HOBr = 20:1. Residual HOBr was quenched after different reaction times (60 and 360 s), by transferring 2 mL of the reaction solution to a volumetric flask, to which 3 mL of an ABTS solution (130 µM ABTS; 40 mM H_2SO_4) was previously added. The absorption was measured at λ = 405 nm and converted to a HOBr concentration (based on a HOBr-ABTS calibration; ε = 30226 M^{-1} cm^{-1}). [28] Figure S21 shows a plot of ln of the relative residual concentration of HOBr as a function of time. The slope of the straight line corresponds to the pseudo first-order rate constant. Division of the pseudo first-order rate constant by the BOC_2O concentration results in the second-order rate constant of the HOBr-BOC_2O reaction (Table S9).

Figure S21 (blue dotted line) shows that the rate of the HOBr decrease is initially higher, which points to an impurity that reacts faster with HOBr than BOC_2O. The determined second-order rate constant for the HOBr-BOC_2O reaction is 10.7 M^{-1} s^{-1} (blue dotted line, 0-60 s, Figure S21) and 5.0 M^{-1} s^{-1} (orange dotted line, 0-360 s, Figure S21). Based on this data set, it can be concluded that BOC_2O is not relevant for HOBr consumption for the experimental conditions in this study with a ca. 10-fold excess relative to *N*-acetylated-Se-amino acid concentrations as the reactivity of studied *N*-acetylated-Se-amino acids towards HOBr exceeds the HOBr-BOC_2O reactivity by several orders of magnitude.

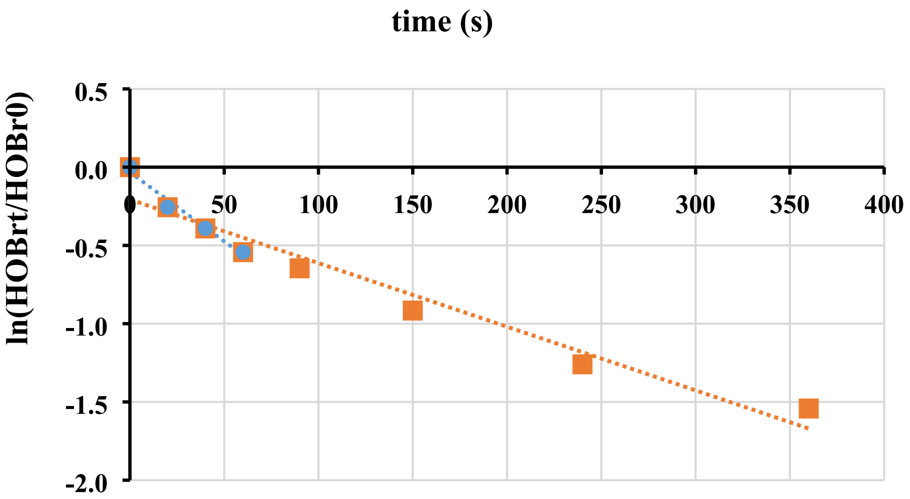

Figure S21: Kinetics of the HOBr- BOC_2O reaction (pseudo-first order conditions, see Table S9) at pH 8. The decrease of the ln of the relative residual concentration of HOBr is illustrated for two time periods of the same experiment: 0-60 s (blue fit) and 0-360 s (orange fit). The experimental conditions are indicated in Table S9.

Table S9: Experimental conditions of the HOBr-BOC_2O kinetic experiment and the determined apparent second-order rate constants at pH 8.

BOC_2O-concentration [µM]	825
HOBr initial [µM]	41.7
PO_4-buffer [mM]	20
pH	8.0
Slope (i.e., $k'_{app,\ pH8\ BOC2O+HOBr}$) 0-60s [$s^{-1}$]	0.0088
Slope (i.e., $k'_{app,\ pH8\ BOC2O+HOBr}$) 0-360s [$s^{-1}$]	0.0041
Second-order rate constant ($k''_{app,\ pH8\ BOC2O+HOBr}$) 0-60s [$M^{-1}\ s^{-1}$]	**10.7**
Second-order rate constant ($k''_{app,\ pH8\ BOC2O+HOBr}$) 0-360s [$M^{-1}\ s^{-1}$]	**5.0**

Text S11: Stoichiometry for reactions between diselenide compounds and HOBr

The stoichiometries of the HOBr-target compound reactions were determined by experiments using an understoichiometric molar concentration of HOBr relative to the target compound concentration. The consumed HOBr is plotted against the consumed target compound and shown in Figure S22. The stoichiometry of reactions between HOBr and diselenides (DMDSe, DPDSe, *N*-acetylated-$SeCys_2$) is close to 3:1.

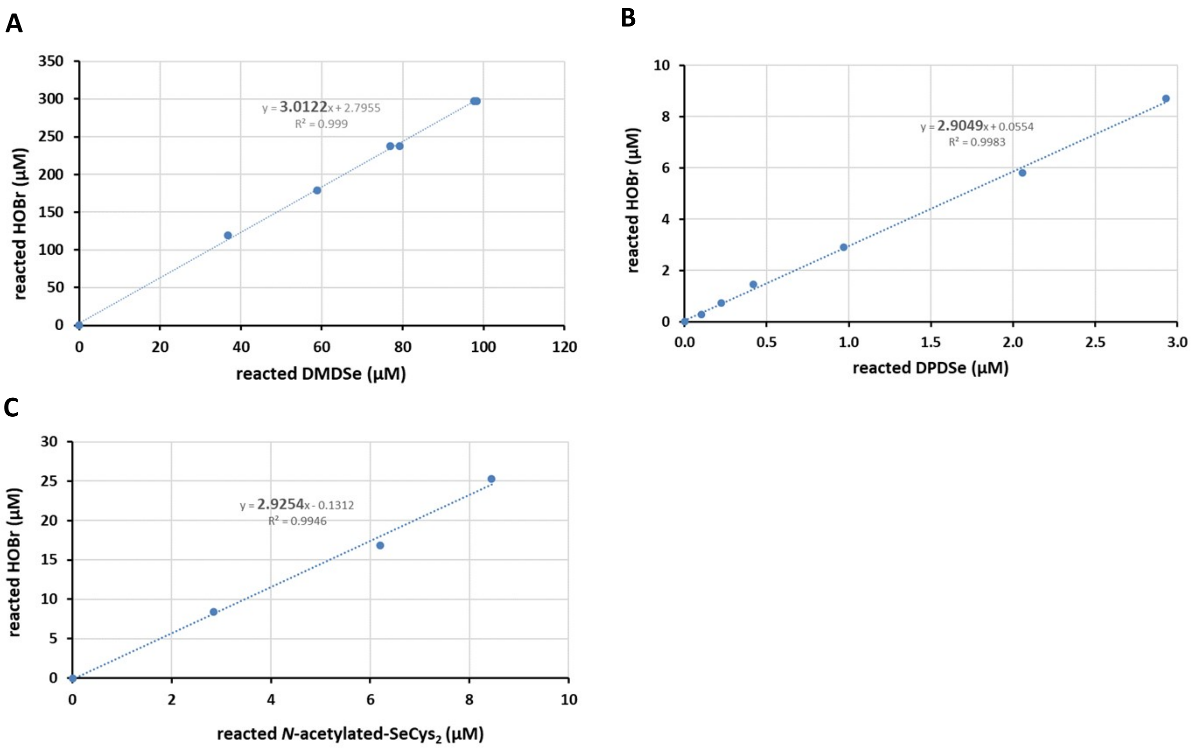

Figure S22: Stoichiometries of reactions between diselenides and HOBr at pH 8 (n=1). (A) DMDSe, (B) DPDSe, (C) *N*-acetylated-SeCys$_2$.
Conditions: pH 8, Buffer media: $[PO_4]_{tot}$ = 20 mM for experiments with DMDSe, $[PO_4]_{tot}$ = 10 mM for experiments with DPDSe and *N*-acetylated-SeCys$_2$. Initial Se-compound concentrations: $[DMDSe]_0$ = 125 µM, $[DPDSe]_0$ = 2.93 µM, [*N*-acetylated-SeCys$_2$]$_0$ = 8.45 µM. HOBr concentrations were varied between 0 and 2.5 times the DMDSe concentration (3 times excess used for DPDSe and *N*-acetylated-SeCys$_2$).

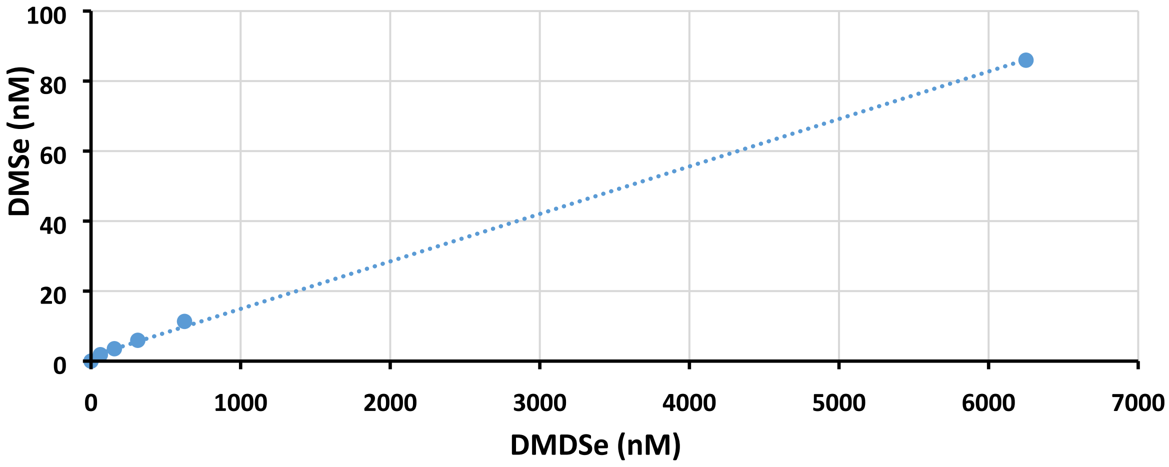

Figure S23: Measured DMS in DMDSe samples via DI-SPME-GC/MS. The slope of 0.0136 corresponds to 1.36 nM DMS in 100 nM DMDSe.

References:

1. Obata, T.; Araie, H.; Shiraiwa, Y., Bioconcentration mechanism of selenium by a coccolithophorid, Emiliania huxleyi. *Plant and cell physiology* **2004,** *45*, (10), 1434-1441.
2. Larsen, E. H.; Hansen, M.; Fan, T.; Vahl, M., Speciation of selenoamino acids, selenonium ions and inorganic selenium by ion exchange HPLC with mass spectrometric detection and its application to yeast and algae. *Journal of Analytical Atomic Spectrometry* **2001,** *16*, (12), 1403-1408.
3. Fan, T. W.; Lane, A. N.; Higashi, R. M., Selenium biotransformations by a euryhaline microalga isolated from a saline evaporation pond. *Environmental science & technology* **1997,** *31*, (2), 569-576.
4. Gómez-Jacinto, V.; García-Barrera, T.; Garbayo, I.; Vílchez, C.; Gómez-Ariza, J. L., Metallomic study of selenium biomolecules metabolized by the microalgae Chlorella sorkiniana in the biotechnological production of functional foods enriched in selenium. *Pure and Applied Chemistry* **2012,** *84*, (2), 269-280.
5. Wrench, J., Selenium metabolism in the marine phytoplankters Tetraselmis tetrathele and Dunaliella minuta. *Marine Biology* **1978,** *49*, (3), 231-236.
6. Bottino, N. R.; Banks, C. H.; Irgolic, K. J.; Micks, P.; Wheeler, A. E.; Zingaro, R. A., Selenium containing amino acids and proteins in marine algae. *Phytochemistry* **1984,** *23*, (11), 2445-2452.
7. Hu, M.; Yang, Y.; Martin, J.-M.; Yin, K.; Harrison, P., Preferential uptake of Se (IV) over Se (VI) and the production of dissolved organic Se by marine phytoplankton. *Marine Environmental Research* **1997,** *44*, (2), 225-231.
8. Amouroux, D.; Pécheyran, C.; Donard, O. F., Formation of volatile selenium species in synthetic seawater under light and dark experimental conditions. *Applied Organometallic Chemistry* **2000,** *14*, (5), 236-244.
9. Kiene, R. P.; Williams, L. P. H.; Walker, J. E., Seawater microorganisms have a high affinity glycine betaine uptake system which also recognizes dimethylsulfoniopropionate. *Aquatic microbial ecology* **1998,** *15*, (1), 39-51.
10. Brock, N. L.; Citron, C. A.; Zell, C.; Berger, M.; Wagner-Döbler, I.; Petersen, J.; Brinkhoff, T.; Simon, M.; Dickschat, J. S., Isotopically labeled sulfur compounds and synthetic selenium and tellurium analogues to study sulfur metabolism in marine bacteria. *Beilstein journal of organic chemistry* **2013,** *9*, (1), 942-950.
11. Van Fleet-Stalder, V.; Chasteen, T. G.; Pickering, I. J.; George, G. N.; Prince, R. C., Fate of Selenate and Selenite Metabolized byRhodobacter sphaeroides. *Appl. Environ. Microbiol.* **2000,** *66*, (11), 4849-4853.
12. Ansede, J. H.; Yoch, D. C., Comparison of selenium and sulfur volatilization by dimethylsulfoniopropionate lyase (DMSP) in two marine bacteria and estuarine sediments. *FEMS microbiology ecology* **1997,** *23*, (4), 315-324.
13. Dickschat, J. S.; Zell, C.; Brock, N. L., Pathways and substrate specificity of DMSP catabolism in marine bacteria of the Roseobacter clade. *ChemBioChem* **2010,** *11*, (3), 417-425.
14. Gabel-Jensen, C.; Lunøe, K.; Gammelgaard, B., Formation of methylselenol, dimethylselenide and dimethyldiselenide in in vitro metabolism models determined by headspace GC-MS. *Metallomics* **2010,** *2*, (2), 167-173.
15. Kumar, K.; Margerum, D. W., Kinetics and mechanism of general-acid-assisted oxidation of bromide by hypochlorite and hypochlorous acid. *Inorganic Chemistry* **1987,** *26*, (16), 2706-2711.
16. McCurry, D. L.; Quay, A. N.; Mitch, W. A., Ozone promotes chloropicrin formation by oxidizing amines to nitro compounds. *Environmental science & technology* **2016,** *50*, (3), 1209-1217.
17. Eaton, A. D.; Glesceri, L.; Greenberg, E. *Standard methods for the examination of water and wastewater. New York*; APHA-AWWA-WEF: 1998.
18. Hach Chlorine, Free and Total. https://www.google.ch/url?sa=t&rct=j&q=&esrc=s&source=web&cd=&ved=2ahUKEwim7JqQ8aDsAhVUTcAKHdaiCVkQFjABegQIAxAC&url=http%3A%2F%2Fwww.hach.com%2Fasset-get.download-en.jsa%3Fid%3D7639984177&usg=AOvVaw1fgO-EnvUbrRQ38rXgVGec

19. Heeb, M. B.; Criquet, J.; Zimmermann-Steffens, S. G.; von Gunten, U., Oxidative treatment of bromide-containing waters: formation of bromine and its reactions with inorganic and organic compounds--a critical review. *Water Res* **2014,** *48*, 15-42.
20. Heeb, M. B.; Kristiana, I.; Trogolo, D.; Arey, J. S.; Von Gunten, U., Formation and reactivity of inorganic and organic chloramines and bromamines during oxidative water treatment. *Water research* **2017,** *110*, 91-101.
21. Criquet, J.; Rodriguez, E. M.; Allard, S.; Wellauer, S.; Salhi, E.; Joll, C. A.; Von Gunten, U., Reaction of bromine and chlorine with phenolic compounds and natural organic matter extracts–Electrophilic aromatic substitution and oxidation. *Water research* **2015,** *85*, 476-486.
22. Troy, R. C.; Margerum, D. W., Non-metal redox kinetics: Hypobromite and hypobromous acid reactions with iodide and with sulfite and the hydrolysis of bromosulfate. *Inorganic Chemistry* **1991,** *30*, (18), 3538-3543.
23. Vriens, B.; Mathis, M.; Winkel, L. H.; Berg, M., Quantification of volatile-alkylated selenium and sulfur in complex aqueous media using solid-phase microextraction. *Journal of Chromatography A* **2015,** *1407*, 11-20.
24. Hubaux, A.; Vos, G., Decision and detection limits for calibration curves. *Analytical chemistry* **1970,** *42*, (8), 849-855.
25. Dodd, M. C.; Buffle, M.-O.; Von Gunten, U., Oxidation of antibacterial molecules by aqueous ozone: moiety-specific reaction kinetics and application to ozone-based wastewater treatment. *Environmental science & technology* **2006,** *40*, (6), 1969-1977.
26. Brenton, A. G.; Godfrey, A. R., Accurate mass measurement: terminology and treatment of data. *Journal of the American Society for Mass Spectrometry* **2010,** *21*, (11), 1821-1835.
27. Loos, M.; Gerber, C.; Corona, F.; Hollender, J.; Singer, H., Accelerated isotope fine structure calculation using pruned transition trees. *Analytical chemistry* **2015,** *87*, (11), 5738-5744.
28. Pinkernell, U.; Nowack, B.; Gallard, H.; Von Gunten, U., Methods for the photometric determination of reactive bromine and chlorine species with ABTS. *Water Research* **2000,** *34*, (18), 4343-4350.
29. Beckwith, R. C.; Wang, T. X.; Margerum, D. W., Equilibrium and kinetics of bromine hydrolysis. *Inorganic chemistry* **1996,** *35*, (4), 995-1000.
30. Liu, Q.; Margerum, D. W., Equilibrium and kinetics of bromine chloride hydrolysis. *Environmental science & technology* **2001,** *35*, (6), 1127-1133.
31. Sivey, J. D.; Arey, J. S.; Tentscher, P. R.; Roberts, A. L., Reactivity of BrCl, Br(2), BrOCl, Br(2)O, and HOBr toward dimethenamid in solutions of bromide + aqueous free chlorine. *Environ Sci Technol* **2013,** *47*, (3), 1330-8.
